# RBCK1 regulates the progression of ER-positive breast cancer through the HIF1α signaling

**DOI:** 10.1038/s41419-022-05473-6

**Published:** 2022-12-06

**Authors:** Zhiguo Niu, Jianing Fan, Fengzhe Chen, Huijie Yang, Xin Li, Ting Zhuang, Chunlei Guo, Qi Cao, Jian Zhu, Hui Wang, Qingsong Huang

**Affiliations:** 1 Henan Key Laboratory of Immunology and Targeted Drugs, School of Laboratory Medicine, Xinxiang Medical University, Xinxiang, Henan Province PR China; 2 Xinxiang Key Laboratory of Tumor Migration and Invasion Precision Medicine, Xinxiang Medical University, Xinxiang, Henan Province PR China; 3 Centre for Transplant and Renal Research, Westmead Institute for Medical Research, The University of Sydney, Sydney, NSW Australia; 4 Department of General Surgery, The Second Hospital, Cheeloo College of Medicine, Shandong University, Shandong, Shandong Province PR China

**Keywords:** Cell migration, Breast cancer

## Abstract

Breast cancer is the most common malignancy in women on a global scale. It can generally be divided into four main categories, of which estrogen receptor ER-positive breast cancer accounts for most breast cancer cases. RBCK1 protein is an E3 ubiquitin ligase containing the UBL, NZF, and RBR domains. It is well known to exhibit abnormal expression in breast tumors, making it a valuable diagnostic marker and drug target. Additionally, studies have confirmed that in breast cancer, about 25 to 40% of tumors appear as visible hypoxic regions, while in hypoxia, tumor cells can activate the hypoxia-inducing factor HIF1 pathway and widely activate the expression of downstream genes. Previous studies have confirmed that in the hypoxic environment of tumors, HIF1α promotes the remodeling of extracellular matrix, induces the recruitment of tumor-associated macrophages (TAM) and immunosuppression of allogeneic tumors, thereby influencing tumor recurrence and metastasis. This research aims to identify RBCK1 as an important regulator of HIF1α signaling pathway. Targeted therapy with RBCK1 could be a promising treatment strategy for ER-positive breast cancer.

## Introduction

Breast cancer is the first female malignancy with worldwide incidence. According to 2022 Cancer Statistics data, breast cancer accounts for about 31% of tumor incidence in women and 15% of tumor-related deaths worldwide [1]. The onset of breast cancer is related to factors such as age >50 years, late menopause, early menarche, and family history of breast cancer [2]. Additionally, atypical hyperplasia of breast lobules and intraepithelial neoplasia are precancerous lesions. A small proportion of breast cancer has a familial predisposition, and genetic mutations such as BRCA1, BRCA2, and ATM can significantly increase the incidence of this type of cancer [3]. Breast cancer is most commonly classified by four molecular classes: Luminal A, Luminal B, HER2, and Triple Negative Breast Cancer [4]. Luminal A and B are both estrogen receptor-positive and can be effectively treated by blocking the estrogen signaling pathway with endocrine therapy [5]. Simultaneously, ER-positive breast cancer accounts for most breast cancers, while triple-negative breast cancer is clinically temporarily controlled by chemotherapy. However, regardless of medical treatment or surgery, most breast cancer patients cannot avoid tumor progression and recurrence [6]. Recurrence and metastasis of tumors is the most important cause of death from breast cancer.

Numerous studies have confirmed that abnormalities and distortions in the structure of blood vessels in tumors lead to poor blood supply and an anaerobic state of tumors [7]. And some tumor cells such as cancer stem cells (CSCs) exhibit enhanced stem cells and activated differentiation potential in the hypoxic tumor microenvironment [8]. In breast cancer, roughly 25 to 40% of tumors present as visible hypoxic areas, and the partial pressure level of oxygen in breast cancer is only one-thirtieth of benign breast tumors [9]. In hypoxic states, tumor cells can activate hypoxia-inducing factor pathways and extensively activate the expression of downstream genes. The hypoxia-inducible factor HIF1 is composed of two subunits, HIF1α and HIF1β. Among them, HIF1β is continuously expressed, while the protein level of HIF1α is precisely regulated by oxygen concentration [10]. The hypoxia-inducing factor HIF1α has been revealed in previous studies to play a key role in tumor recurrence and metastasis [11, 12]. High expression of HIF1α predicts early recurrence and metastasis of breast cancer and is inversely correlated with survival in patients [13, 14]. HIF1α is a transcription factor consisting of 836 amino acids. Among them, the bHLH part is responsible for DNA binding, the PAS part is responsible for the assembly of heterodimers of HIF1β, and its NTAD and CTAD domains are involved in mediating post-translational regulation, transcriptional activity regulation, and stability regulation of HIF1α protein [15, 16]. At normal oxygen concentrations, proline hydroxylase (PHDs) can pass via the P402 and P564 sites of hydroxylated HIF1α. Subsequently, hydroxylated HIF1α can be identified and degraded by the VHL protein (Von Hippel-Lindau) [17]. Additionally, hydroxylation at the N803 site can block the interaction between HIF1α and transcriptional activator P300, thereby blocking the activation of HIF1α to downstream genes [18]. Therefore, the half-life of HIF1α at normal oxygen concentrations is only about 5 to 15 min [19]. In hypoxia, the hydroxylation of proline and aspartic acid of HIF1α is inhibited, resulting in the increased stability of the HIF1α protein [20]. HIF1α then interacts with transcriptional co-activators and binds to transcriptional response elements on DNA, resulting in the expression of pro-tumor progression and metastasis genes, including GΜLT-1 and VEGFA [21–23]. Therefore, the activity of the information pathway of HIF1α is primarily controlled by protein stability under oxygen dependence. However, in the environment of extensive hypoxia in cancer tumors, the stability of HIF1α protein was significantly improved, and the oxygen-dependent regulation method was greatly weakened. Therefore, some oxygen-independent protein stability regulation methods may play a key role in regulating the stability of HIF1α protein and the strength of information pathways in tumors.

Previous studies have displayed that the RBCK1 protein regulates the mechanism by which estrogen signaling pathways mediate breast cancer proliferation and progression [24]. The degree of expression of RBCK1 in breast tumors increased significantly compared to healthy breast epithelial cells. RBCK1 protein was associated with resistance to endocrine therapy in breast cancer [25]. The study has empirically confirmed that RBCK1 protein positively correlates with estrogen receptor expression. Phenotypic studies of cells report that silencing RBCK1 significantly inhibits the proliferation of breast cancer cells.

This study investigates the regulatory mechanism of RBCK1 on the HIF1α signaling pathway and the novel concept of the development of ER-positive breast cancer. RBCK1, a regulatory factor, may become a hidden target for ER-positive breast cancer treatment. It is also a novel concept for the clinical follow-up development of drugs treating ER-positive breast cancer.

## Results

### In hypoxic conditions, RBCK1 can accelerate the migration and cloning capacity of ER-positive breast cancer cells, as well as energy metabolism levels

**Fig. 1:**
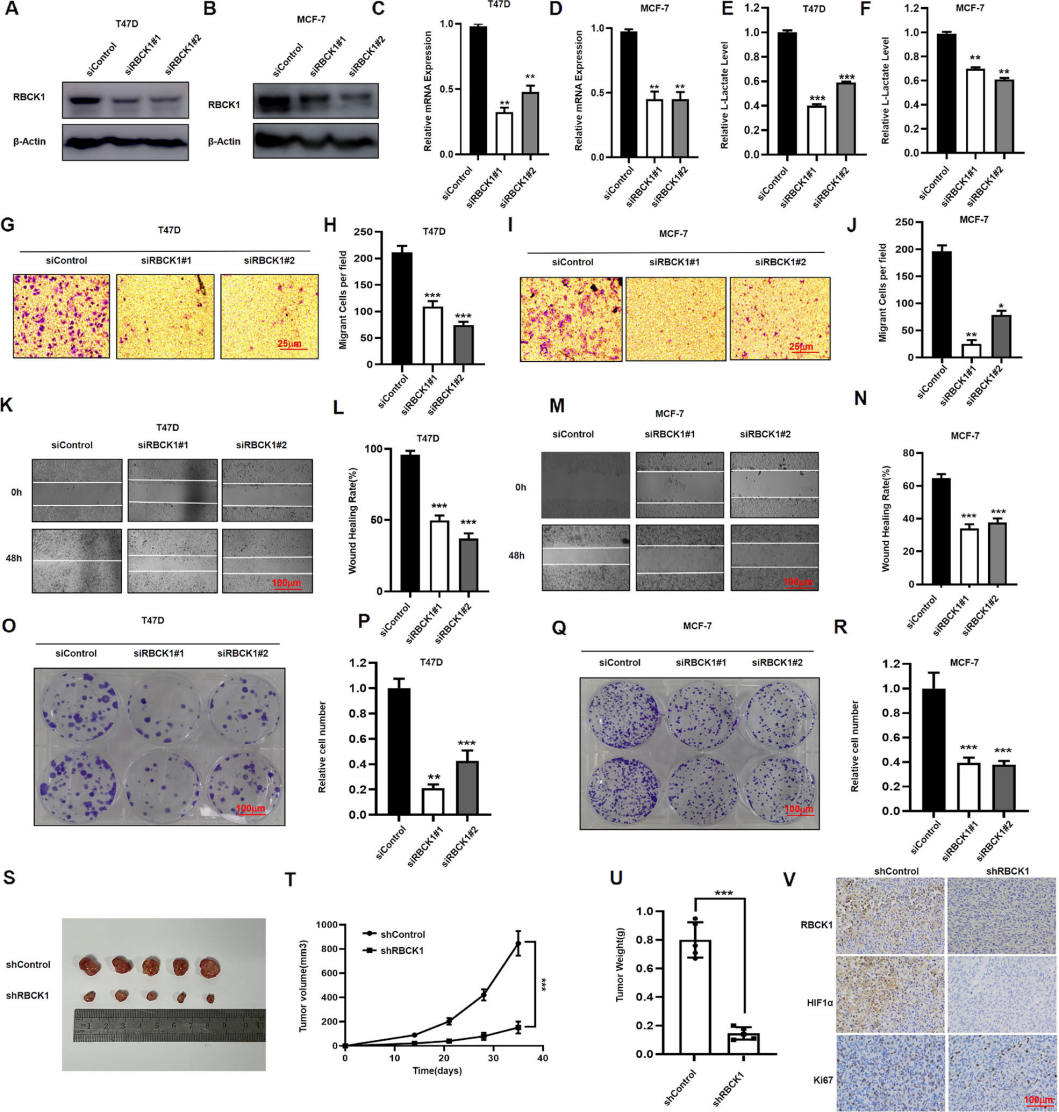
RBCK1 depletion inhibits migration in ER-positive breast cancer cells. **A**–**D** RBCK1 was silenced in T47D and MCF-7, and its silencing effect was then detected by western blot technology and real-time PCR technology. **E**, **F** RBCK1 depletion under hypoxia reduced the level of energy metabolism in T47D and MCF-7 cells, which were transfected with siControl or siRBCK1. After 36 h, the cells were treated with hypoxia for 12 h, L-lactate level was determined by L-Lactate Assay Kit. **G**–**J** RBCK1 knockdown inhibits ER+ cell migration in T47D and MCF-7 cells under hypoxia. We planted cells in a transwell chamber and looked under the microscope at the migration capacity of cells. We then counted and recorded the images obtained by ImageJ and then analyzed them with Prism 8.0, *p* < 0.05, which is statistically significant (*p < 0.05; **p < 0.01; ***p < 0.001). **K**–**N** RBCK1 promotes migration in ER+ breast cancer cells under hypoxia. Wound healing assay under hypoxia of T47D and MCF-7 cells were transfected with siControl or siRBCK1. The experiment should be repeated more than three times so that the results obtained are statistically significant. **O**–**R** RBCK1 depletion inhibits the ability of colony formation in T47D and MCF-7 cells under hypoxia. Crystal violet staining assay evaluated the ability of colony formation of MCF-7 and T47D cells with the silence of RBCK1, and the quantitative analysis is displayed (right). The data are presented by the means ± SD (*n* = 3). (*p < 0.05; **p < 0.01; ***p < 0.001). **S**–**U** RBCK1 knockdown inhibits tumor growth of T47D cells in xenografted models. After the tumor is planted in mice, the growth status of mouse transplant tumor is monitored. After five weeks, mice are sacrificed, and measurements and follow-up experiments are performed. *p < 0.05, **p < 0.01, ***p < 0.001 for student’s t-test. **V** IHC staining shows that the expression of Ki67 and HIF1α decreased after RBCK1 knockdown. IHC staining of Ki67, RBCK1, and HIF1α in xenograft model tumor.

To determine whether RBCK1 is a promoter or inhibitor in ER-positive breast cancer, we used ER-positive breast cancer cell lines, T47D and MCF-7, as part of our experiments. We obtained six pairs of RBCK1 siRNAs from Jima Genetics. To ensure the accuracy of the experiment, we tested their silencing efficiency by western blotting and real-time PCR technology and then took the two most pronounced siRNAs (Fig. 1A–D). Next, these two siRNAs of RBCK1 were applied to observe their effect on the phenotype of ER-positive breast cancer cells under hypoxic conditions. Cell trans-well experiments suggest that silencing RBCK1 can inhibit cell migration in T47D and MCF-7 (Fig. 1G–J). Wound healing experiments show that after silencing RBCK1, the healing capacity of cells weakens (Fig. 1K–N). We also silenced RBCK1 in T47D and MCF-7 and detected that the clonal formation capacity of cells was inhibited (Fig. 1O–R). Therefore, we observe that knocking down RBCK1 in ER-positive breast cancer cells can inhibit the migration of ER-positive breast cancer cells. We also discovered in T47D and MCF-7 that silencing of RBCK1 can potentially mitigate cell lactate levels (Fig. 1E, F). Since the phenotype experiments have revealed that silencing RBCK1 can inhibit the migration, healing, colony formation, and lactate metabolism levels of T47D and MCF-7, we performed in vivo tumor growth experiments. The results indicate that RBCK1 knockdown can significantly limit the growth of mouse transplant tumors, including the weight of tumors and volume (Fig. 1S–U). The same is true for immunohistochemical results (Fig. 1V), from which it can be concluded that silencing RBCK1 can inhibit tumor progression in ER-positive breast cancer cells under hypoxic conditions.

### RBCK1 regulates the progression of ER-positive breast cancer by the HIF1α signaling pathway

**Fig. 2:**
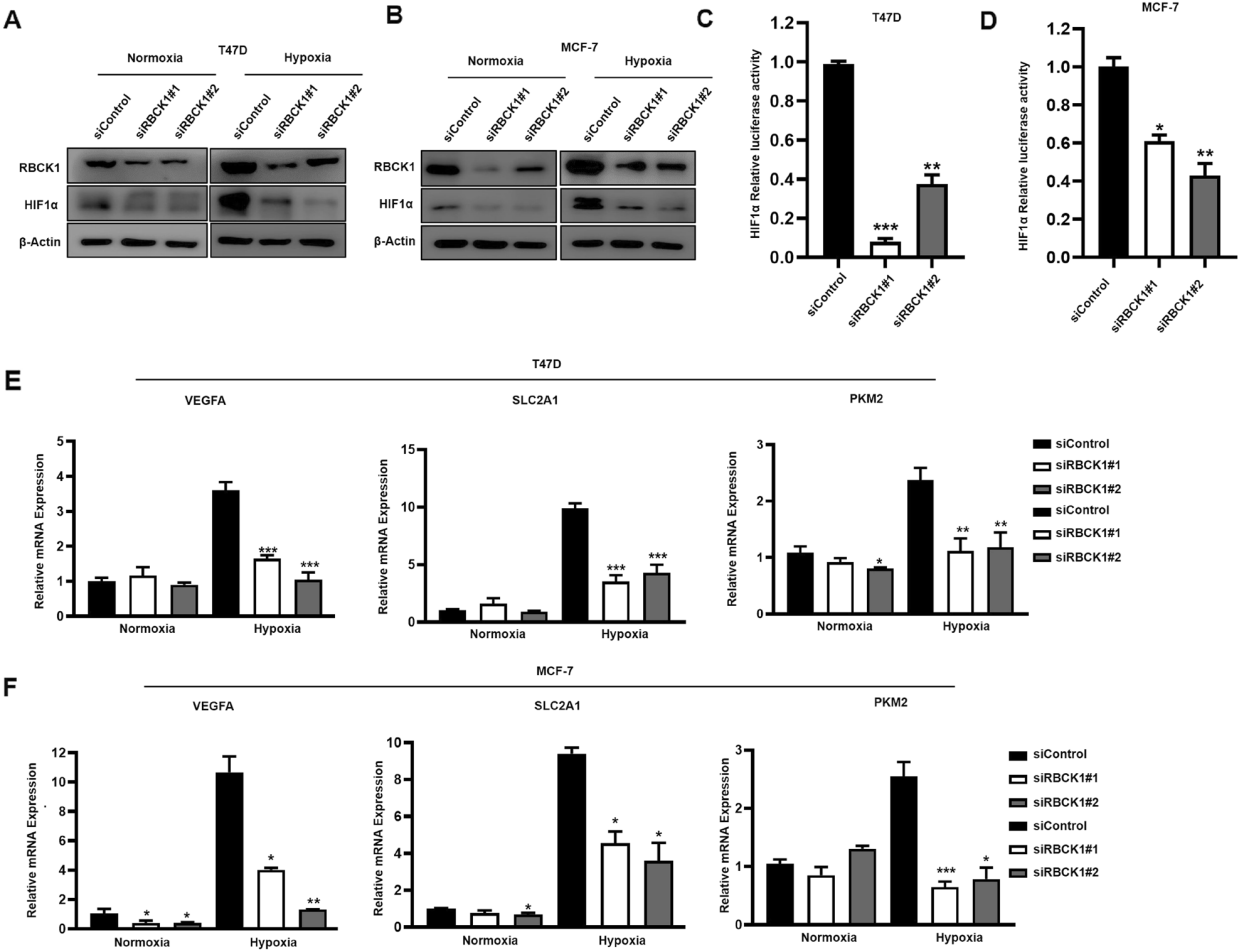
RBCK1 depletion decreases HIF1α protein level and HIF1α target gene expression in ER+ breast cancer cells. **A**, **B** RBCK1 depletion under hypoxia decreases HIF1α protein level more significantly than that under normoxia in T47D and MCF-7 cells. Transfection of siControl or siRBCK1 in T47D and MCF-7. After 36 h, the cells were cultivated under hypoxia or normoxia for 12 h; we lysed and collected cell proteins for western blot analysis. The protein levels of RBCK1 and HIF1α were detected by western blot analysis. β-Actin is the internal reference used in the experiment. The experiment should be repeated more than three times, implying that the obtained results are statistically significant. *p < 0.05, **p < 0.01, ***p < 0.001 for student’s t-test. **C**, **D** RBCK1 exhaustion decreases HIF1α luciferase activity in T47D and MCF-7 cells. Transfection of siControl or siRBCK1 in T47D and MCF-7. After 24 h of transfection, the cells were transfected with HIF1α luciferase reporter plasmid and Renilla plasmid. After 48 h, the cells were lysed and collected, and luciferase activity was detected. **E**, **F** RBCK1 depletion decreases HIF1α target gene expression. T47D and MCF-7 cells were transfected with siControl or siRBCK1. After 36 h, the cells were cultivated under hypoxia or normoxia for 12 h, and total RNA was extracted for gene expression analysis. By real-time quantitative fluorescence PCR technology, the mRNA expression of the target gene downstream of HIF1α in different groups was detected, and the internal reference was 36B4. The experiment should be repeated more than three times, and the results comparison standard is as follows: *p < 0.05; ** *p* < 0.01; ***p < 0.001.

**Fig. 3:**
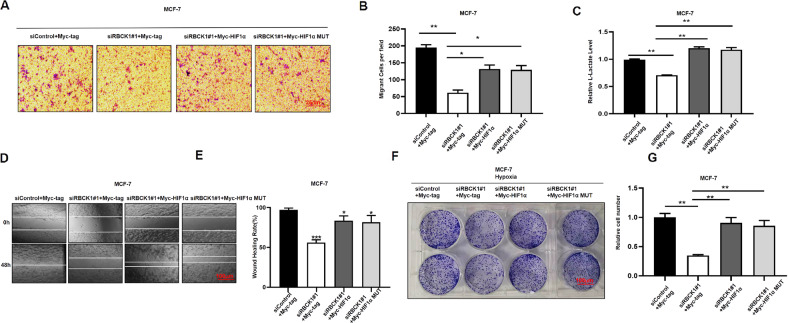
RBCK1 promotes the migration of ER+ breast cancer cells through HIF1α signaling. **A**, **B** RBCK1 depletion decreased the migration capacity of ER+ breast cancer cells, which can be rescued after HIF1α WT or HIF1α MUT overexpression. The migration capacity of tumor cells after RBCK1 knockdown was detected by trans-well technology, imaging was performed by microscopy, and finally, count analysis was conducted. The experiment should be repeated more than three times, and the results comparison standard is as follows: *p < 0.05; **p < 0.01; ***p < 0.001. **C** L-Lactate Assay proved that RBCK1 depletion decreased the level of energy metabolism of ER+ breast cancer cells, which can be rescued after HIF1α WT or HIF1α MUT overexpression. The data are represented in means ± SD. *p < 0.05, **p < 0.01, ***p < 0.001 for student’s t-test. **D**, **E** Wound healing assay proved that the migration capacity of ER+ breast cancer cells was decreased because of RBCK1 depleting, while this could be reversed after HIF1α WT or HIF1α MUT overexpression. Wound closure is measured at different time points and analyzed computationally. The data are represented in means ± SD. *p < 0.05, **p < 0.01, ***p < 0.001 for student’s t-test. **F**, **G** RBCK1 depletion decreased the ability of colony formation of ER+ breast cancer cells, which can be reversed after HIF1α WT or HIF1α MUT overexpression. The results are then counted and analyzed using ImageJ. The data are represented in means ± SD. *p < 0.05, **p < 0.01, ***p < 0.001 for student’s t-test.

We explored the role of RBCK1 on the HIF1α signaling pathway. After the transfection of siRBCK1 in T47D and MCF-7 by a western blot and real-time PCR techniques, the detected expression of HIF1α protein (Fig. 2A, B) and its classic downstream genes such as VEGFA and SLC2A1 were significantly downregulated (Fig. 2E, F). After arriving at the above conclusion, we carried out the investigation and then verified whether RBCK1 can have an impact on the transcriptional level of HIF1α. Through the luciferase reporter gene experiment, it was revealed that when RBCK1 was silenced in T47D and MCF-7. The transcriptional activity of HIF1α was also significantly reduced (Fig. 2C, D). These results show that RBCK1 can affect its gene levels by influencing the transcriptional activity of HIF1α. According to the results obtained, it can be concluded that, on the one hand, RBCK1 can promote tumor progression, such as migratory cloning of ER-positive breast cancer; on the other hand, RBCK1 can impact the activity of the hypoxia-inducible factor HIF1α signaling pathway by increasing the expression of HIF1α. Therefore, we hypothesize that RBCK1 promotes tumor progression in ER-positive breast cancer by the HIF1α pathway. We performed a rescue experiment to assess the idea, and after silencing RBCK1 of MCF-7, its migration capacity by the trans-well experiment is weakened, and this condition can be rescued by the overexpression of HIF1α (Fig. 3A, B). Through scratch experiment detection, it was proven that the healing ability of tumor cells could also be saved (Fig. 3D, E). The clone formation experiment also detected similar results (Fig. 3F, G). Additionally, the lactate detection experiments have also displayed that the lactate metabolism level of MCF-7, which was transfected with siRBCK1, can also be reversed (Fig. 3C). We used a mutant plasmid of HIF1α, which does not change the function of the original HIF1α but strengthens its stability, avoiding the instability of experimental results due to the short half-life of the original HIF1α. The results were consistent with those obtained prior. In summary, it can be demonstrated that RBCK1 promotes tumorigenesis and progression of ER-positive breast cancer by influencing the HIF1α signaling pathway.

### RBCK1 is elevated in breast cancer and positively correlates with the HIF1α signaling pathway in MCF-7 samples

**Fig. 4:**
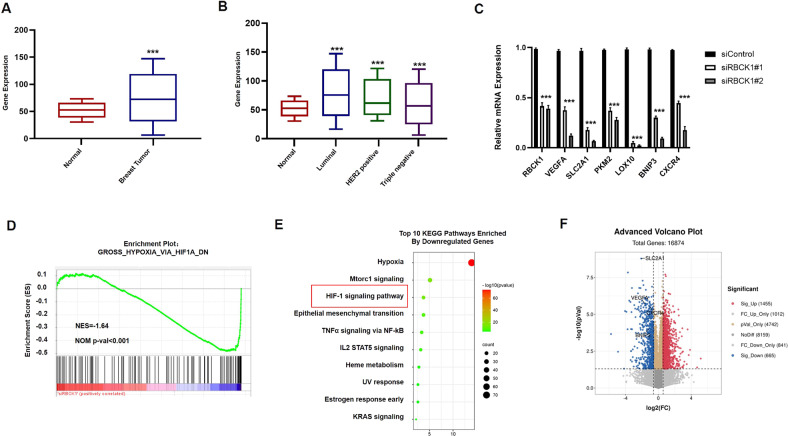
Bioinformatic analysis reveals the correlation between RBCK1 and HIF1α signaling in ER-positive breast cancer cells. **A**, **B** Publicly available data shows that RBCK1 is significantly highly expressed in ER-positive breast cancer. (http://oncomine.org). **C**–**E** Gene Set Enrichment Analysis and KEGG map of RNA sequencing data display that the silence of RBCK1 in MCF-7 inhibits HIF1α signaling pathway. MCF-7 cells were transfected with siRBCK1 or siControl. After 36 h, the cells were treated with hypoxia for 12 h, then the total mRNA of the two groups of cells was extracted for real-time PCR technology and RNA sequencing analysis. The siControl and siRBCK1 groups were analyzed more than three times. **F** Volcano map shows that after RBCK1 level is knocked down in MCF-7, the mRNA levels of HIF1α target genes VEGFA, SLC2A1, and BNIP3 are reduced.

We obtained the expression of RBCK1 in various types of breast cancer in the recognized TCGA database. As can be seen, compared with healthy tissues, RBCK1 is significantly higher in breast cancer patient specimens compared to other molecular types of breast cancer samples, whereby RBCK1 is expressed more in ER-positive breast cancer (Fig. 4A, B, https://www.oncomine.org). Next, we transfected siRBCK1/siControl in MCF-7, ER-positive breast cancer, and after 36 h, the cells were hypoxic for 12 h. The total RNA was then extracted for qPCR and RNA sequencing. From the qPCR analysis results (Fig. 4C), we selected a group of siRBCK1#2 with good silencing efficiency of RBCK1 for RNA sequencing (triplicate analysis of siControl and siRBCK1). We analyzed the RNA SEQ results (GSE196274). The gene set enrichment analysis plot and KEGG plot demonstrate that RBCK1 consumption in MCF-7 breast cancer cells inhibited the HIF1α signaling pathway and positively correlated with the HIF1α signaling pathway (Fig. 4D, E). The volcano map demonstrates the HIF1α classical target genes VEGFA, SLC2A1, and BNIP3, whose expression was significantly reduced due to RBCK1 knockdown in MCF-7 cells (Fig. 4F). These results demonstrated that RBCK1 was highly expressed in ER-positive breast cancer cells and positively correlated with HIF1α.

### RBCK1 binds to HIF1α, mainly through the RBR domain of RBCK1 and the N-terminal region of HIF1α

**Fig. 5:**
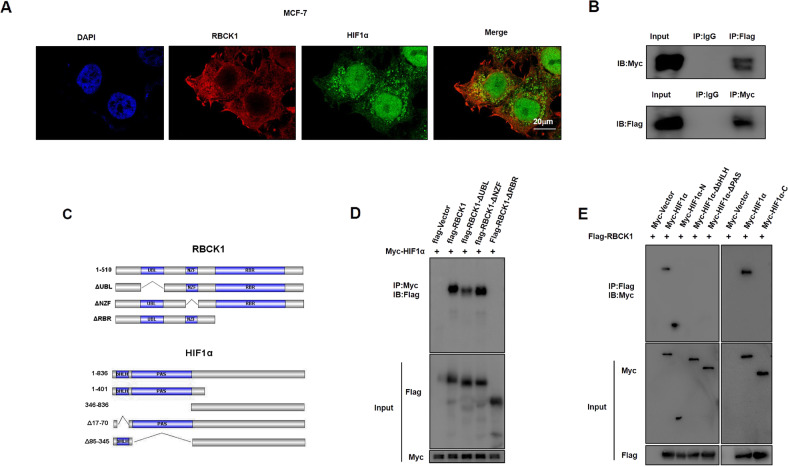
RBCK1 associates with HIF1α N-terminal through its RBR domain. **A** Intracellular localization analysis of RBCK1 and HIF1α by immunofluorescence assay. Immunofluorescence experiments are not started until MCF-7 is in good condition. This figure illustrates the intracellular localization of MCF-7, where HIF1α is green, and RBCK1 is red. The nucleus is stained blue by DAPI. **B** Co-IP assay revealed an association between RBCK1 and HIF1α in HEK293T cells. Flag-RBCK1 and Myc-HIF1α were co-transfected in HEK293T, and HEK293T cells were then harvested with RIPA lysis buffer. Co-IP was performed using an antibody as indicated. Co-IP experiments have demonstrated a correlation between RBCK1 and HIF1α in HEK293T cells. **C** The figure indicates the full length of RBCK1 and HIF1α and the domains of each part. **D** RBCK1 interacts with HIF1α through its RING domain. In HEK293T, the full length of Myc-HIF1α and Flag-RBCK1 and their missing mutant plasmids (ΔUBL, ΔNZF, and ΔRBR) are transferred. After 24 h of cell transfection, MG132 is added to HEK293T for 6 h. Subsequently, the cells are lysed with IP lysate, and the cell proteins are collected for IP and western blot experiments. Simultaneously, we used anti-Myc and anti-Flag antibodies to detect the specific domain of RBCK1 interaction with HIF1α. **E** The N-terminal is essential for HIF1α to interact with RBCK1. In HEK293T, the full length of Flag-RBCK1 and Myc-HIF1α and their deletion of mutant plasmids (N-terminal, C-terminal, ΔbHLH, and ΔPAS) are co-transfected. After 24 h of cell transfection, MG132 is added to HEK293T for 6 h. Subsequently, the cells are lysed with IP lysate, and the cell proteins are collected for IP and western blot experiments. Simultaneously, we used anti-Myc and anti-Flag antibodies to detect the specific domain of HIF1α interaction with RBCK1.

To gain further insights into the mechanism of action of RBCK1 in the HIF1α signaling pathway, we investigated the subcellular localization of RBCK1 in ER-positive breast cancer cells MCF-7. Immunofluorescence experiments show the presence of HIF1α in both the cytoplasm and the nucleus, and RBCK1 is explicitly seen to be less in the nucleus compared to its cytoplasm, but they are both in the cytoplasm (Fig. 5A). Therefore, their interaction was detected by Co-IP experiments in HEK293T (Fig. 5B). We then needed to further derive a specific combination, so we constructed the truncated plasmid of RBCK1 and HIF1α plasmids, and the domain of RBCK1 primarily has the three domains of UBL, NZF, and RBR. The domain of HIF1α contains the N-terminus, C-terminus, bHLH, PAS domain (Fig. 5C). Next, our IP technique detected that the interaction between RBCK1 and HIF1 α was primarily the RBR domain of RBCK1 and the N-terminal of HIF1α (Fig. 5D, E).

### RBCK1 indirectly inhibits the ubiquitination degradation of HIF1α at the K48 ubiquitination site, thereby promoting its protein stability, and the RBR domain of RBCK1 plays an integral role in it

**Fig. 6:**
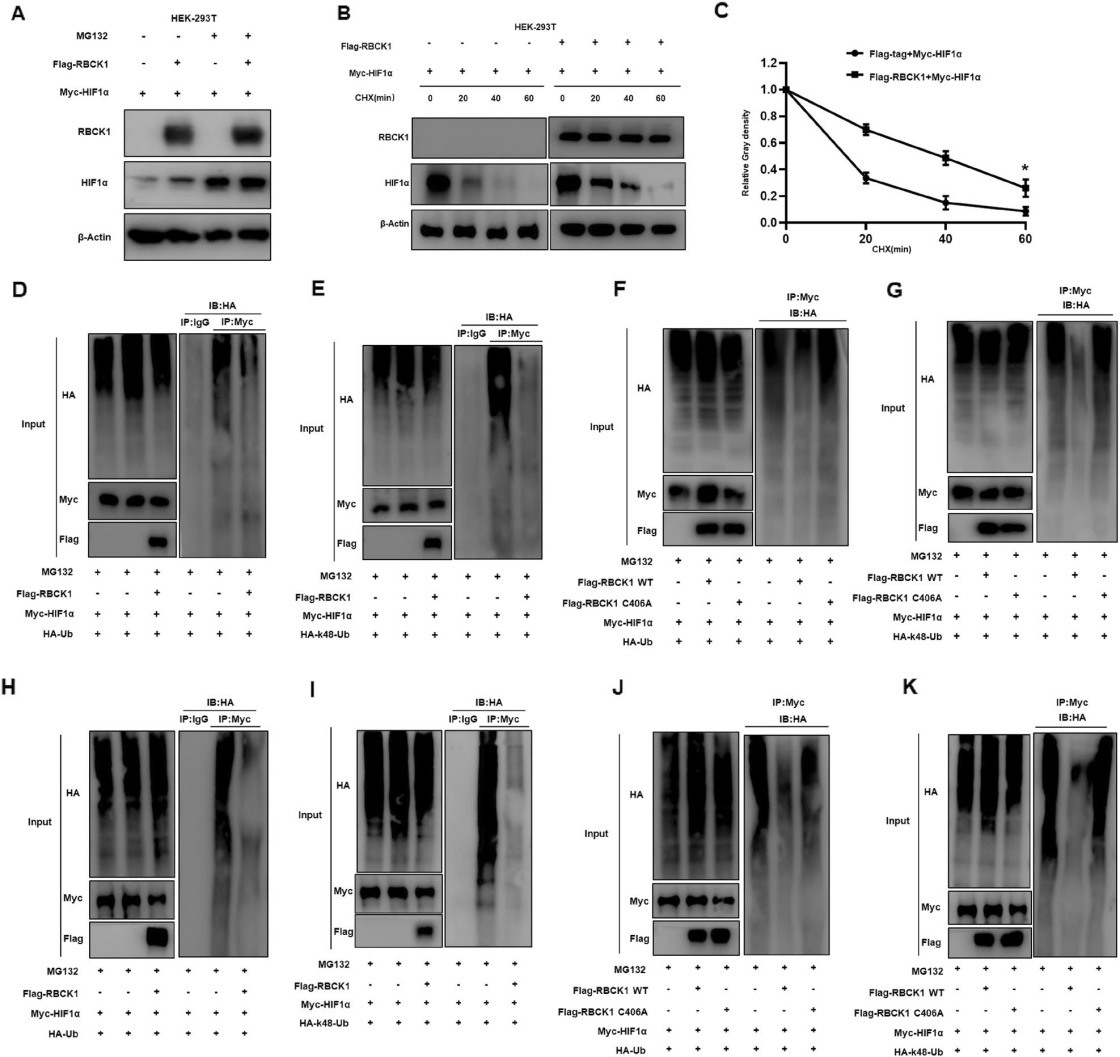
RBCK1 inhibits HIF1α K48-linked polyubiquitination and degradation. **A** The proteasome inhibitor MG132 can reverse the increased stability of HIF1α due to RBCK1 overexpression. Myc-HIF1α and Flag-tag/Flag-RBCK1 plasmids were transferred in HEK293T, and MG132 was added to the cells for 6 h after transfection. The cells are lysed with RIPA, the protein is collected, and changes in the protein level of HIF1α are detected by western blot. The experiment results were repeated at least three times to reach the above. **B**, **C** RBCK1 raised HIF1α half-life in HEK293T cells. Myc-HIF1α and Flag-tag/Flag-RBCK1 were co-transferred to HEK293T to detect the effect of RBCK1 on the half-life of HIF1α. After 24 h of transfection, the protein synthesis inhibitor cycloheximide is added to the cells at different times to inhibit the production of new HIF1α proteins. Subsequently, the cells are lysed with RIPA, the cell proteins are collected, and the western blot is performed to detect changes in the protein levels of HIF1α. Finally, the result is quantified and analyzed graphically using ImageJ software. The experiment was repeated at least three times to reach the above results. **D** RBCK1 decreases the total polyubiquitination of HIF1α. In HEK293T, HIF1α, HA-Ubi, and Flag-tag/Flag-RBCK1 are transferred to form a ubiquitination system. After 24 h of transfection, the cells are lysed with RIPA, and cellular proteins are collected for ubiquitination IP and protein electrophoresis. Detecting the ubiquitination effect of RBCK1 on HIF1α by anti-HA antibody. **E** RBCK1 reduces K48-linked polyubiquitination of HIF1α. In HEK293T, HIF1α, HA-K48 Ubi, and Flag-tag/Flag-RBCK1 are transferred to form a ubiquitination system. After 24 h of transfection, the cells are lysed with RIPA, and cellular proteins are collected for ubiquitination IP and protein electrophoresis. RBCK1 to HIF1α ubiquitination sites are detected by anti-HA antibodies. **F**, **G** RBCK1 inhibits HIF1α polyubiquitination, where RBCK1’s ubiquitin ligase functional RING domain plays a crucial role. In HEK293T co-transferred HIF1α, HA-Ubi/HA-K48 Ubi, and Flag-tag/Flag-RBCK1 WT/Flag-RBCK1 C406A (RING MUT) plasmids, RIPA lysed and obtained the cell protein after 24 h of transfection. Subsequently, in western blot and IP experiments, changes in HIF1α ubiquitination were detected by anti-HA antibodies. **H** RBCK1 decreases the total polyubiquitination of HIF1α. In MCF-7, HIF1α, HA-Ubi, and Flag-tag/Flag-RBCK1 are transferred to form a ubiquitination system. After 24 h of transfection, the cells are lysed with RIPA, and cellular proteins are collected for ubiquitination IP and protein electrophoresis. Detecting the ubiquitination effect of RBCK1 on HIF1α by anti-HA antibody. **I** RBCK1 reduces K48-linked polyubiquitination of HIF1α. In MCF-7, HIF1α, HA-K48 Ubi, and Flag-tag/Flag-RBCK1 are transferred to form a ubiquitination system. After 24 h of transfection, the cells are lysed with RIPA, and cellular proteins are collected for ubiquitination IP and protein electrophoresis. RBCK1 to HIF1α ubiquitination sites are detected by anti-HA antibodies. **J**, **K** RBCK1 inhibits HIF1α polyubiquitination, where RBCK1’s ubiquitin ligase functional RING domain plays a crucial role. In MCF-7 co-transferred HIF1α, HA-Ubi/HA-K48 Ubi, and Flag-tag/Flag-RBCK1 WT/Flag-RBCK1 C406A (RING MUT) plasmids, RIPA lysed and obtained the cell protein after 24 h of transfection. Subsequently, in western blot and IP experiments, changes in HIF1α ubiquitination were detected by anti-HA antibodies.

We then further explored the specific mechanism of the interaction between RBCK1 and HIF1α, considering that RBCK1 is an E3 ubiquitin ligase, so we performed a series of protein stability experiments. Overexpression of RBCK1 can significantly increase the protein expression of HIF1α. However, when we added MG132 stimulation, the protein level of HIF1α was not affected by whether RBCK1 was overexpressed (Fig. 6A), suggesting that RBCK1 may regulate HIF1α levels through the ubiquitination-proteasome pathway. Subsequently, we found that overexpression of RBCK1 can increase the protein stability of HIF1α by extending the half-life of the HIF1α protein (Fig. 6B, C). The above suggests that RBCK1 may regulate the expression of HIF1α through the ubiquitination-proteasome pathway, so we then used the ubiquitination IP experiment to detect the effect of RBCK1 on HIF1α ubiquitination. The results demonstrate that RBCK1 can inhibit polyubiquitination HIF1α (Fig. 6D). Therefore, we need to explore further how RBCK1 affects the ubiquitination of HIF1α. The results show that RBCK1 inhibits the ubiquitination degradation of HIF1α by using the K48 ubiquitination site (Fig. 6E). We then mutated the RING domain in the RBR of RBCK1 and constructed the Flag-RBCK1 C406A plasmid. Through ubiquitin IP experiments, it was revealed that the RBR domain of RBCK1 plays an important role in this process (Fig. 6F, G). To further determine whether this mechanism is established in ER positive breast cancer, we repeated the mechanism experiments in MCF-7 (Fig. 6H–K). These results suggest that there must be another E3 ubiquitin ligase between RBCK1 and HIF1α that mediates the ubiquitination of RBCK1 to HIF1α, meaning that RBCK1 can indirectly regulate HIF1α levels through the ubiquitination-proteasome pathway.

## Discussion

**Fig. 7:**
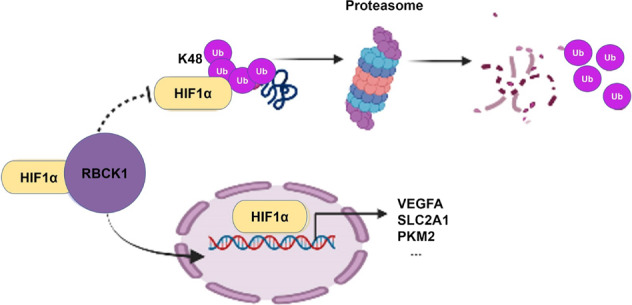
The hypothetical model of the mechanism by which RBCK1 regulates HIF1α signaling in ER+ breast carcinoma. RBCK1 promoted target gene activation of HIF1α pathway and accelerated the progression of ER-positive breast cancer by interacting with HIF1α protein and inhibiting HIF1α ubiquitination at K48 ubiquitination site.

We found a E3 ubiquitin ligase RBCK1, which is prominently elevated in breast cancer and mainly exhibits carcinogenic effects. In ER-positive breast cancer cell lines, the RBCK1 protein and the HIF1α signaling pathway are positively correlated. Therefore, we have made the speculation that RBCK1 promotes the progression of cell migration in the HIF1α signaling pathway and ER-positive breast cancer by promoting the ubiquitination of another ubiquitin ligase, which regulate the ubiquitination of HIF1α, thereby inhibiting the K48 ubiquitination degradation of the HIF1α, and ultimately improving its protein stability (Fig. 7). Since there are many ubiquitinated proteins that can act on HIF1α [26, 27], we need to further explore what this ubiquitinated ligase protein is and the specific mechanism between it, RBCK1 and HIF1α. In any case, our study identified a new regulator of the HIF1α signaling pathway, RBCK1, which can modulate tumor progression in ER-positive breast cancer cells by modulating this pathway, providing a new target for the treatment of ER-positive breast cancer.

Breast cancer occurs in many genetic factors, such as BRCA1/BRCA2 abnormalities [28]. However, most of them are caused by genetic mutations, and oncogenes not only participate in cellular mutations during the initiation phase but also play a vital role after the formulation of breast cancer [29]. Simultaneously, estrogen abnormalities and the occurrence of breast cancer also play a crucial role because estrogen primarily acts on the precipitating stage of cancer formation, which is directly related to human endocrine disorders [30]. The recurrence and metastasis of tumors is the most important cause of death from breast cancer. Over 90% of breast cancer patients die from distant metastases. In breast cancer, about 25 to 40% of tumors present as visible hypoxic areas, and the partial pressure level of oxygen in breast cancer is only one-thirtieth of that of benign breast tumors [31]. In hypoxic states, tumor cells can activate hypoxia-inducing factor pathways and extensively activate the expression of downstream genes. The hypoxia-inducible factor HIF1 is composed of two subunits, HIF1α and HIF1β. Among them, HIF1β is continuously expressed. Meanwhile, the protein level of HIF1α is precisely regulated by oxygen concentration [19]. The hypoxia-inducing factor HIF1α has been depicted in a series of prior works to have a core role in tumor recurrence and metastasis [32, 33].

Prior works have demonstrated that the hypoxia-inducible factor HIF1α can potentially induce the production of TWIST and SNAIL and promote epithelial interstitial transformation [34, 35], while HIF1α can promote extracellular matrix degradation by upregulating the expression of MMP2/MMP9, thus helping tumor cell metastasis [36, 37]. Hypoxia can induce the recruitment of tumor-associated macrophages (TAMs) and immunosuppression of allogeneic tumors [38]. Additionally, HIF1α promotes neovascularization by regulating the upregulation of the target gene VEGFA. GLUT-1 is also upregulated by promoting glucose transport and tumor energy metabolism [39, 40]. And hypoxic cancer cells were shown to release substantial amounts of TF that was mainly associated with secreted microvesicles with exosome-like characteristics [41]. In clinically relevant works in the literature, it was found that the expression level of HIF1α is an important indicator of breast cancer recurrence and metastasis. High expression of HIF1α predicts early recurrence and metastasis of breast cancer, which is inversely correlated with survival [42, 43]. Simultaneously, the expression of the downstream gene of HIF1α was significantly upregulated in triple-negative breast cancer and was associated with prognosis [44]. A series of animal transplant tumor experiments have displayed that silencing the HIF1α pathway inhibits lymph nodes and lung metastases in breast cancer [45]. Unfortunately, despite numerous studies attempting to develop small molecule inhibitors against HIF1α, no mature drugs for HIF1α in clinical oncology treatment are readily available. Further in-depth investigation of the molecular mechanisms of intracellular regulation of the HIF1α pathway will offer new strategies and ideas for treating breast cancer.

RBCK1 (also known as HOIL-1L) containing RANBP2 and C3HC4 zinc finger is a 58 kDa protein that contains the N-terminal ubiquitin-like (UBL) domain, the Npl4 zinc finger (NZF) domain, and the catalytic carbon terminal RBR domain. Many E3 ubiquitin ligases exhibit abnormal expression in tumors, making them valuable diagnostic markers and drug targets. Publicly-available databases have determined that mRNA expression of RBCK1 in breast cancer is substantially higher than that of healthy breast epithelium [46] and that RBCK1 mRNA levels in ER-positive breast cancer tissues were significantly increased compared to ER-negative breast cancer tissues [47]. Prior works have depicted that RBCK1 promotes the proliferation of MCF-7 and T47D breast cancer cells with ER-positive expression and shows that this is attributable to the upregulation of ERα gene and protein expression [48]. It has also been confirmed that high expression of RBCK1 is closely associated with cancer-associated fibroblasts (CAFs) in colorectal cancer and tumor-associated macrophages in kidney cancer [49, 50]. Various studies have shown that RBCK1 may affect the tumor microenvironment by enriching CAF, adipocytes, endothelial cells, TAM, etc., thereby exerting its carcinogenic role. This also provides new ideas for our follow-up research. According to the results, it was found that RBCK1 can interact with HIF1α protein in ER-positive breast cancer cells, increasing its protein stability. RBCK1 modulates the HIF1α signaling pathway through a post-translational mechanism influencing breast cancer development. Our results offer a novel idea for this concept. RBCK1, which belongs to the E3 ubiquitin ligase protein, is likely to become a new target for treating ER-positive breast cancer.

In summary, a hypothetical model of RBCK1 modulating the HIF1α signaling mechanism in ER+ breast cancer: RBCK1 can interact with the HIF1α protein to indirectly inhibit its polyubiquitination and degradation at K48 site, thereby promoting HIF1α target gene expression as well as breast cancer progression.

## Materials and methods

### Cell culture

The cell lines used in this experiment primarily include T47D and MCF-7, which belong to the ER+ breast cancer cell line, and HEK293T, all of which were obtained from the American Type Culture Collection (ATCC). All cells were cultured at 37 °C, 5% CO_2_ in a cell culture incubator. The culture medium used in cell culture was prepared by mixing high-sugar DMEM (DMEM, D6429, Sigma-Aldrich) with 10% fetal bovine serum nutrients (FBS, 10270-106, Gibco) and 1% penicillin antibiotic (Beyotime). All cell lines were certified by cell line authentication via Short Tandem Repeat (STR), which was performed via the PowerPlex 21 system. We found that the STR data of T47D, MCF-7, and HEK293T cell lines were consistent with STR data in ATCC.

### Plasmids and siRNA

The Myc-HIF1α plasmid and the Flag-RBCK1 plasmid were acquired from the Origene Company (https://www.origene.com). The deletion mutants of RBCK1 and HIF1α were respectively sub-cloned from the full-length plasmid of RBCK1 and HIF1α. Simultaneously, the HA-Ub, HA-K48, and HA-K63 plasmids used in the experiments were purchased from the companies mentioned above. To knock down the RBCK1 level in the cells, we purchased six pairs of siRNAs from Gene Pharma and screened for the two with the optimal silencing effect. The transfection reagents applied during the experiment were Lipofectamine 2000 (1662298, Invitrogen) and Lipofectamine™ RNAi-MAX (13778150, Invitrogen). The RBCK1 siRNA sequences were: siRNA#1 GCCUCAGCUACCAUGCATT dTdT; UGCAAUGGUAGCUGAAGGCTT and siRNA#2 CACACCUUCUGCAGGGAGUTT dTdT; ACUCCCUGCAGAAGGUGUGTT. In transplant tumor animal experiments, we constructed a stable cell line of RBCK1 knockdown in T47D, and we initially co-transferred pMD2G, psPAX2, and shRBCK1 in HEK293T, in 48 h. We infected the T47D ER+ breast cancer cells with the resulting viral supernatant and then obtained RBCK1 stably knocked down T47D ER+ breast cancer cells. The system contained either an RBCK1 knockdown sequence (shRBCK1) or a negative control sequence (shcontrol). The shRNA sequences were as follows: 5′- CCCTGAGGATTACAGCGATT-3′, Negative control: 5′-UUCUCCGAACGUGUCACGU-3′.

### RNA extraction and qPCR analysis

We applied the Trizol method (15596-026, Invitrogen) to extract total RNA according to the operating procedures provided by the reagent manufacturer. Based on the real-time PCR method, the reverse transcription was performed with Takara reverse transcriptase (RR036A, Takara). A total of 2 µg purified RNA was utilized to synthesize cDNA and then amplified by PCR using specific primers. Then, RT-PCR was performed using an ABI7500 real-time fluorescence quantitative PCR system and SYBR Green (A25742, Thermo Fisher). The internal reference we applied was 36B4. The primer sequence is illustrated in Table 1. We used the 2^−ΔΔCT^ method to detect relative gene expression levels. We repeated this at least three times for every experiment.

**Table 1:** The primer sequence of qPCR analysis.

Primer	Sequence
36B4 F	GGCGACCTGGAAGTCCAACT
36B4 R	CCATCAGCACCACAGCCTTC
RBCK1 F	TGCTCAGATGCACACCGTC
RBCK1 R	CAAGACTGGTGGGAAGCCATA
VEGFA F	AGGGCAGAATCATCACGAAGT
VEGFA R	AGGGTCTCGATTGGATGGCA
SLC2A1 F	TCTGGCATCAACGCTGTCTT
SLC2A1 R	AGCCAATGGTGGCATACACA
PKM2 F	CTCCTTCAAGTGCTGCAGTG
PKM2 R	GGCCTTGCCAACATTCATGG
CXCR4 F	CCATTCCTTTGCCTCTTTTGC
CXCR4 R	TGACCAGGATGACCAATCCA
LOX10 F	CCAGCAGATCCAATGGGAGAAC
LOX10 R	ATCAGCAGGATCGGAGTGCG
BNIP3 F	ATGTCGCAGAACGGAGCG
BNIP3 R	TAGAAACCGAGGCTGGAACG

### Wound healing assay

Cells were treated first, and then pancreatic enzymes digested the resuspended cells and evenly spread them into 6 or 12-well plates for transfection after the cells were adherent. After transfection for 24 h, the cell density reached a value above 90%, and scratches were made with the tip of yellow pipette. The wound distance was taken and measured at various time points according to the experimental needs and cell state and normalized with the starting time point. The recovery rate for wound healing: [1−(wound width at a given time/wound width at t = 0)] × 100%.

### Trans-well assay

A 24-well plate was prepared, and a 500 μL 20% serum-containing medium (DMEM + 20% FBS) was added per well. Clean trans-well chambers were placed in the wells, allowing the medium to soak the chamber membrane. A total of 200 μL of serum-free medium suspension containing different groups of cells to the upper layer of the chamber were added, ensuring that the cells were evenly distributed in the chamber membrane at 37 °C. They were cultured in a cell culture incubator for about 16 h. After fixing the cells passing through the membrane at 4% paraformaldehyde, they were stained with crystal violet. Finally, they were imaged under a microscope, and ImageJ was used to analyze the count.

### Western blotting

Cells were treated according to different needs of the experiment and lysed with RIPA (Beyotime) on ice to collect cell proteins. The protein expression of the cells was then detected by western blot technology. The antibodies needed for the proteins detected in this experiment are: anti-RBCK1 (26367-1-AP, Proteintech), anti-HIF1α (SC-135151, Santa Cruz), anti-β-Actin (A5441, Sigma), anti-HA (MMS-101R, Biolegend), anti-Myc (60003-2-lg, Proteintech), and anti-Flag (20543-1-AP, Proteintech). After the protein was electrophoretic, transparabed, and blocked, we incubated the corresponding primary antibody and the secondary antibody of the primary antibody species. Finally, we visualized the fluorescent signal of the resulting protein using AI600 (GE), during which the membrane was pre-processed with an Immobilon Western Chemilum HRP Substrate Kit (Millipore Co, Billerica).

### Luciferase assay

Plasmids such as the HIF1α luciferase reporter gene and Renilla plasmid were transfected in cells according to different experimental needs, followed by a detailed operation according to the instructions of the Dual-Luciferase Reporting Kit (Promega). The luciferase activity was measured by a luminometer microplate reader for various groups of cells. The experimental results were analyzed by Prism 8.0 (GraphPad). The *p* < 0.05 was statistically significant.

### Co-immunoprecipitation assay

Cell proteins were collected with Western and IP lysates. A proteasome inhibitor was also used (ST506 p0013, Beyotime). 12000×G was kept at 4 °C after centrifugation for 30 min, and the collected supernatant was incubated with the required antibody or control IgG and protein A/G agarose (p2051 p2053, Beyotime) at 4 °C overnight. The next day, at 4 °C, 3000 × G thrived thrice through centrifugation for 10 min and rinsed with a lysis buffer (p0013f, Beyotime). The supernatant was discarded. A 2×SDS-PAGE buffer was added and boiled at 99 °C for 10 min. SDS-PAGE electrophoresis was performed. The resulting membrane was then incubated with the corresponding primary antibody overnight at 4 °C. After membrane washing, it was incubated with the secondary antibody of HRP-labeled Goat anti-Mouse/Rabbit IgG (H + L) for 2 h. Finally, we visualized the fluorescent signal of the resulting protein using AI600 (GE), during which the membrane was pre-processed with the Immobilon Western Chemilum HRP Substrate Kit (Millipore Co, Billerica).

### Protein stability assays

Pancreatin digestion resuspends the cells and distributes them evenly onto a 24-well plate. The corresponding plasmids, Flag-RBCK1/Flag-tag and Myc-HIF1α were transfected by different wells. After 48 h of transfection, 100 μM cycloheximide (C7698, Sigma) stimulation was introduced at various time points, and the protein level of HIF1α was detected by a western blot, thereby detecting the effect of RBCK1 on the stability of HIF1α protein.

### Analysis of protein ubiquitination

Pancreatin digestion resuspends HEK293T cells and distributes them evenly onto a 24-well plate. The subsequent transfection of various pores corresponds to plasmids, Flag-RBCK1/Flag-tag, Myc-HIF1α, and HA-ub. A 10 μM MG132 (474 787, Sigma) was added to the cells after 48 h of transfection to stimulate for 6 h and detect the polyubiquitination of the HIF1α protein by a western blot.

### Poly-ubiquitination detection assay

To detect the K48 polyubiquitination of HIF1α in cells, we co-transfected Flag-RBCK1/Flag-tag and Myc-HIF1α and K48-Ub plasmids in HEK293T. 48 h after transfection by a western blot and IP technology, we obtained the corresponding protein supernatant. Finally, we visualized the fluorescent signal of the resulting protein using AI600 (GE), during which the membrane was pre-processed with an Immobilon Western Chemilum HRP Substrate Kit (Millipore Co, Billerica).

### Immunofluorescence assay

The well-treated MCF-7 cells were sequenced in the following order. First, 4% paraformaldehyde (p0099, Beyotime) underwent treatment for 20 min and was washed thrice with PBS. 0.25% Triton X-100 (t8200, Solarbio) was added to the cells, which stood at room temperature for 5 min. A total of 3% BSA (st025, Beyotime) was used to block them for 1 h after washing with PBS. Then, the primary antibody was incubated overnight at 4 °C, and the antibodies used here were: rabbit anti-RBCK1 polyclonal antibody (26367-1-AP, Proteintech) and mouse anti-HIF1α monoclonal antibody (SC-135151, Santa Cruz), followed by Alexa flow 647 (Invitrogen) anti-rabbit antibody. After staining with fluorescent secondary antibodies the next day, stain nuclei with DAPI were used for another 5 min. Negative controls were cultured with secondary antibodies without primary antibodies. Finally, a confocal laser scanning microscope (Leica TCS SP8 STED) was employed for photography, further analysis, and mapping with ImageJ.

### L-Lactate assay

The L-Lactate level of T47D and MCF-7 was detected using an L-Lactate Assay Kit (ab65331, Abcam). The cells were transfected with different plasmids. At 24 h after transfection, lysis was used, and cell supernatant was obtained, followed by measuring and recording the L-Lactate level of the cells according to the kit instruction manual. The obtained results were analyzed using Prism 8.0 (GraphPad).

### Colony formation assays

Plasmids in MCF-7 and T47D cells were transfected according to experimental needs, and pancreatic enzyme digestion resuspended cells to a 6-well plate (2,000 cells per well) 24 h after transfection. Cell culture was preserved for 10–12 days, a new medium was used every two days in between. The cells were subsequently fixed with 4% paraformaldehyde for 20 min and stained with a crystal violet solution for 30 min after three PBS washes. The image was then collected and illustrated using ImageJ.

### Publicly available clinical data analysis

Through the TCGA database, we analyzed the expression levels of RBCK1 in normal breast tissue and various kinds of breast cancer. We also identified the correlation between RBCK1/HIF1α and the prognosis of ER-positive breast cancer in the KMMPLOT database (https://kmplot.com). The results of the analysis were calculated using Prism 8.0 (GraphPad).

### Xenograft tumor model

We prepared multiple five-week-old female BALB/c nude mice and divided them into two groups. shControl or shRBCK1 lentiviral vector transduction of 3 × 10^6^ T47D cells was injected subcutaneously into every mouse independently. The tumor size was measured once every three days. After five weeks, the mice were dislocated and sacrificed, and all tumors were removed, photographed in groups, and then weighed using an electronic scale. The calculation formula of the tumor volume used was: tumor volume = length × width^2^/2.

### Statistics

The data analysis was conducted using GraphPad Prism 8 software and graphing. The student t-test was used to compare the difference between the two groups, and *p* < 0.05 was considered statistically significant (*p < 0.05; **p < 0.01; ***p < 0.001). The Kaplan-Meier method was used for single-gene prognosis analysis.

## Supplementary information

Author Contributions Section

Supplemental Material-WB

Original Data File

A reproducibility checklist that details key elements of the experimental and analytical design of the submission.

## Data Availability

The publicly available data are provided in the supplementary materials. The datasets used and analyzed during the current study are available from the corresponding author on reasonable request.
