# Correction: RBCK1 regulates the progression of ER-positive breast cancer through the HIF1α signaling

**DOI:** 10.1038/s41419-025-08384-4

**Published:** 2026-02-05

**Authors:** Zhiguo Niu, Jianing Fan, Fengzhe Chen, Huijie Yang, Xin Li, Ting Zhuang, Chunlei Guo, Qi Cao, Jian Zhu, Hui Wang, Qingsong Huang

**Affiliations:** 1 Henan Key Laboratory of Immunology and Targeted Drugs, School of Laboratory Medicine, Xinxiang Medical University, Xinxiang, Henan Province China; 2 Xinxiang Key Laboratory of Tumor Migration and Invasion Precision Medicine, Xinxiang Medical University, Xinxiang, Henan Province China; 3 Centre for Transplant and Renal Research, Westmead Institute for Medical Research, The University of Sydney, Sydney, NSW Australia; 4 Department of General Surgery, The Second Hospital, Cheeloo College of Medicine, Shandong University, Shandong, Shandong Province China

Correction to: *Cell Death & Disease* 10.1038/s41419-022-05473-6, published online 06 December 2022

Since the publication of the aforementioned article, our research team identified errors in the data presented in Fig. 1S and Fig. 1M of the paper during a retrospective review of previous studies. The correct data are provided in the email attachments (TIFF and PPT files). These errors do not affect the main conclusions or the original data of the study, as the experimental trends observed upon RBCK1 depletion are consistent between the corrected figures and the erroneous ones. We sincerely apologize for any inconvenience this may have caused. Our team will take this as a serious lesson, rigorously reviewing all previously published and future work to uphold academic integrity.

Figure 1S original
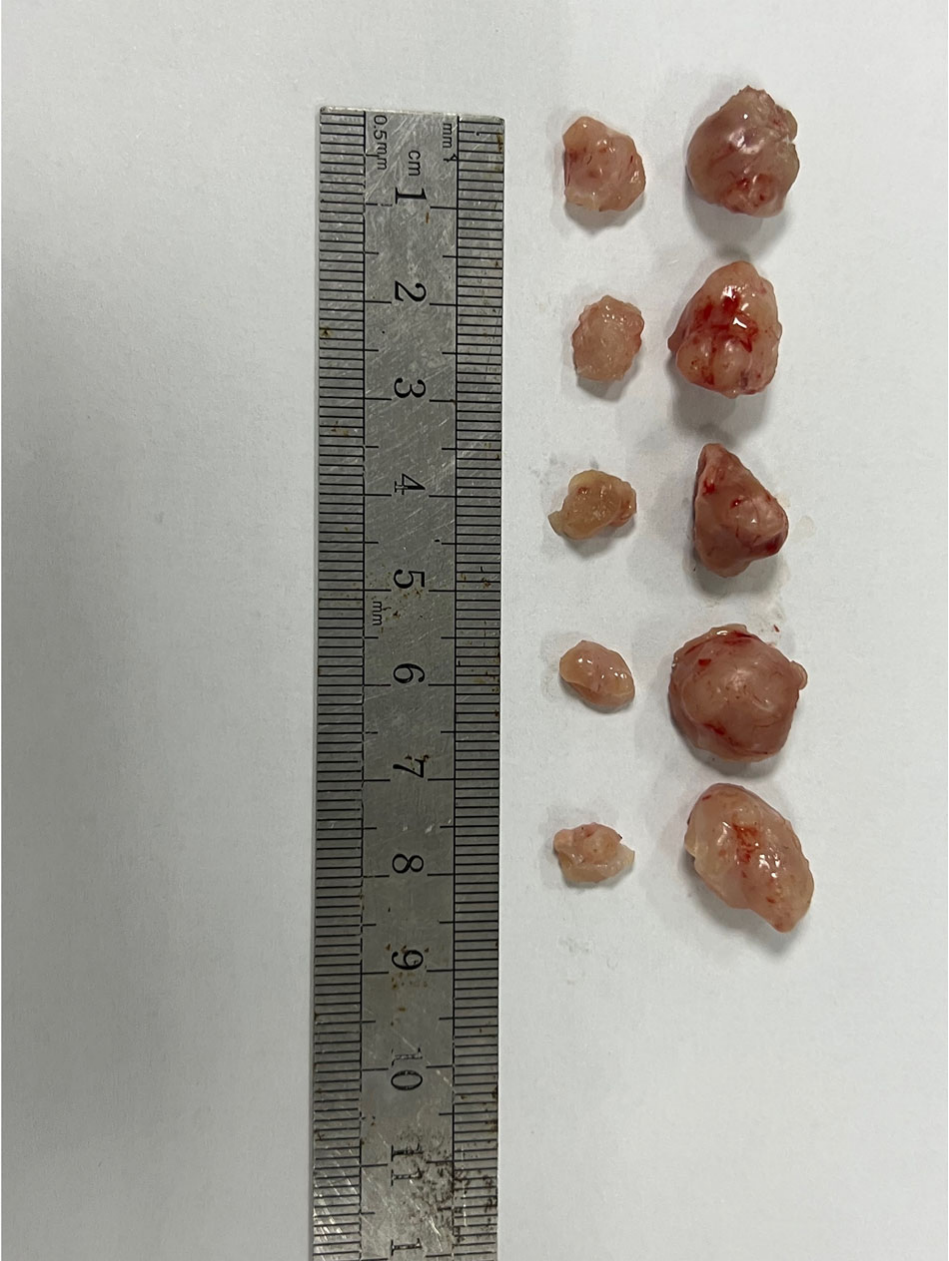


Figure 1S amended
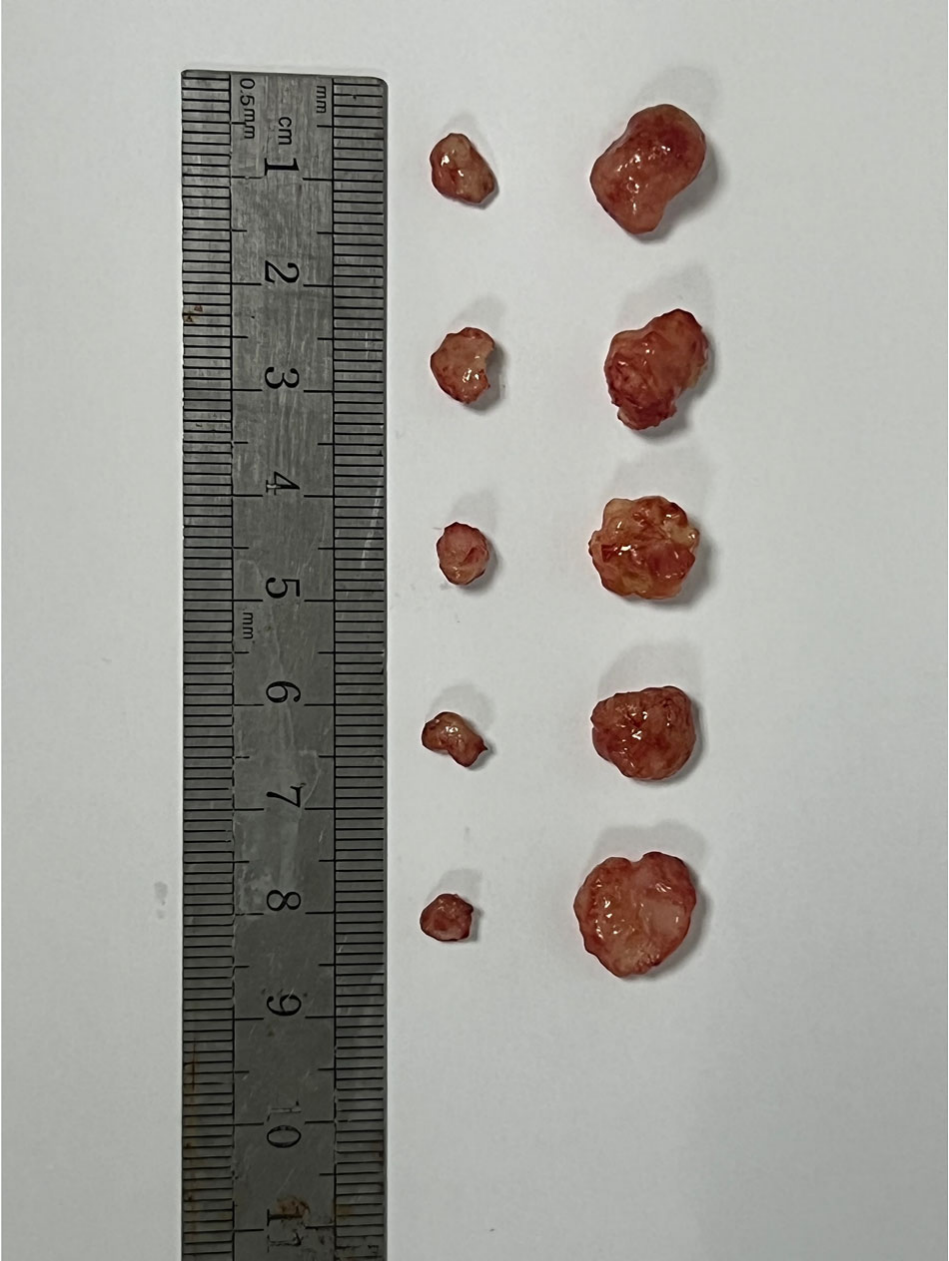


Figure 1M original
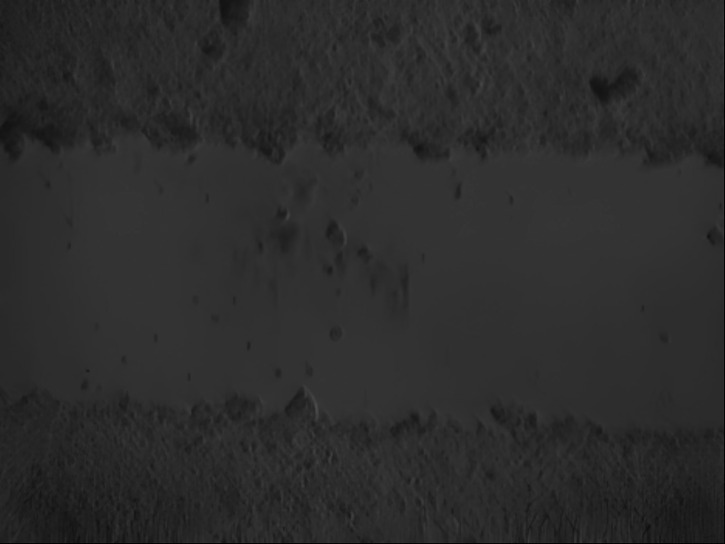


Figure 1M amended
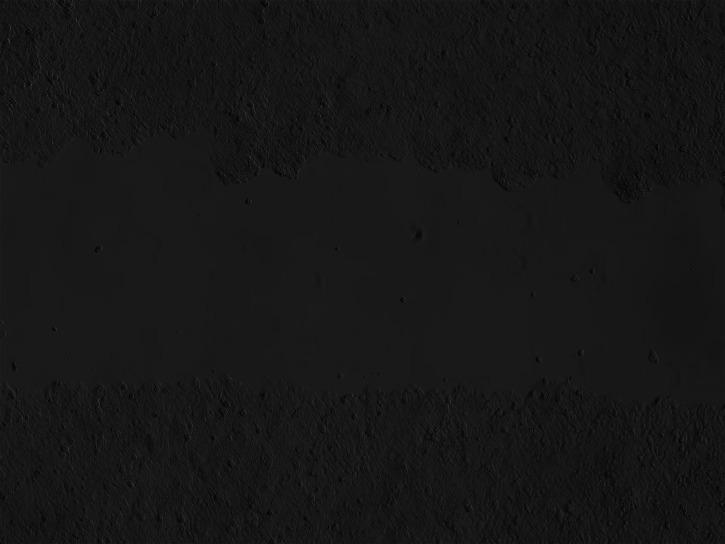


The original article has been corrected.

