# Supplementary material for: RBCK1 regulates the progression of ER-positive breast cancer through the HIF1α signaling
Source: Cell Death Dis. 2022 Dec 6;13(12):1023. doi: 10.1038/s41419-022-05473-6 (PMC9726878; doi:10.1038/s41419-022-05473-6)
Supplement: Supplementary file 1 — Author Contributions Section [file 41419_2022_5473_MOESM1_ESM.docx]

**Author Contributions Section**

QH, HW, and JZ conceived the design of that study. ZN, JF and FC performed the molecular and cellular biology of the study and performed the cellular phenotype assays. HY, XL, and TZ performed the xenograft mice study. CG and QC performed the RNA-sequence data analysis and bioinformatics data analysis. JZ and QH wrote the manuscript and approved the manuscript. QH, ZN, and QC offered funding support and project supervision during the revision stage.
