# Supplementary figures and images for: RBCK1 regulates the progression of ER-positive breast cancer through the HIF1α signaling
Source: Cell Death Dis. 2022 Dec 6;13(12):1023. doi: 10.1038/s41419-022-05473-6 (PMC9726878; doi:10.1038/s41419-022-05473-6)

Figure1  
A-B

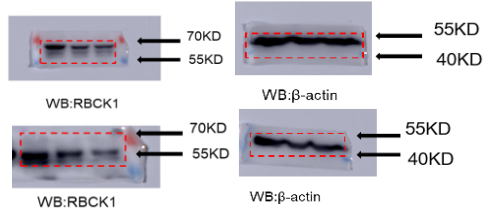

**Figure2A**  
**T47D**

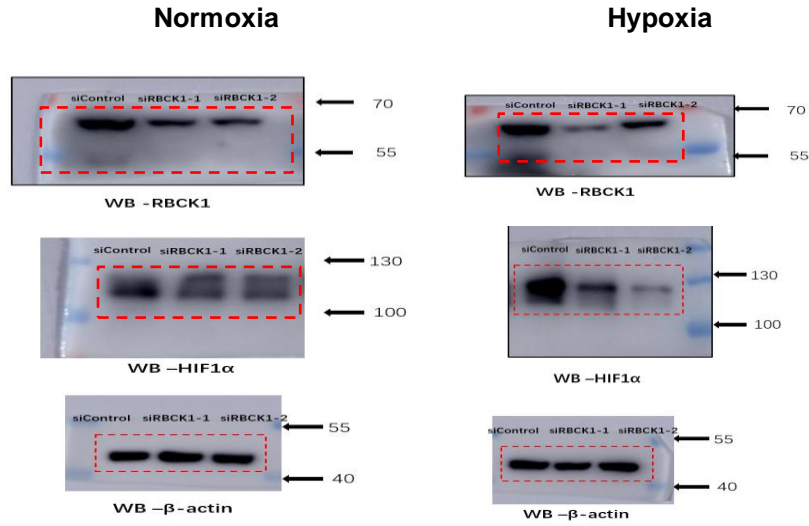

**Figure2B**  
**MCF-7**

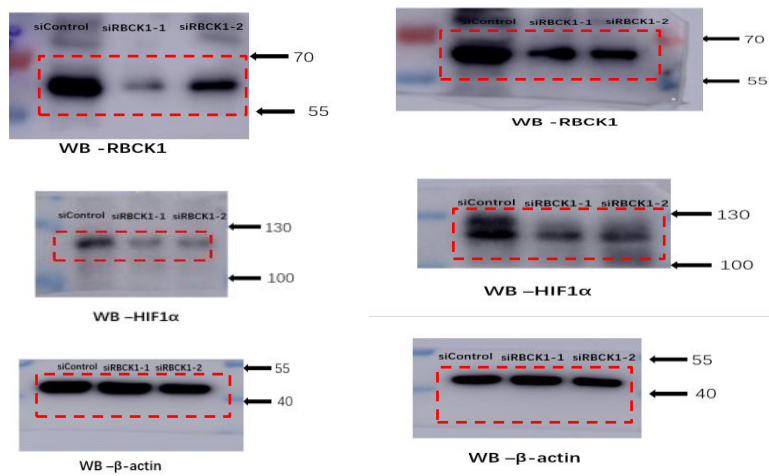

Figure5

B

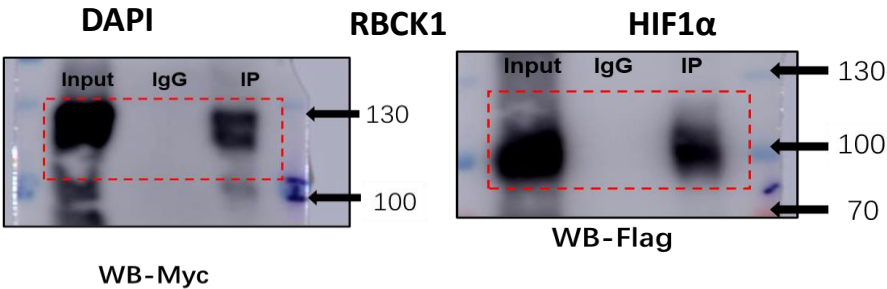

D

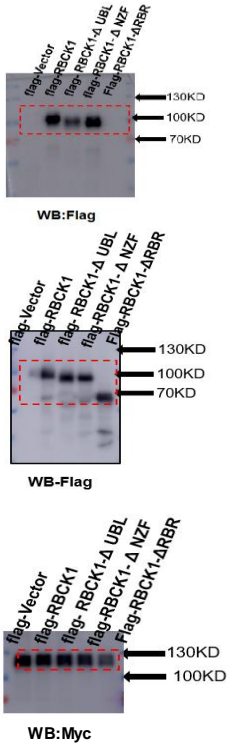

E

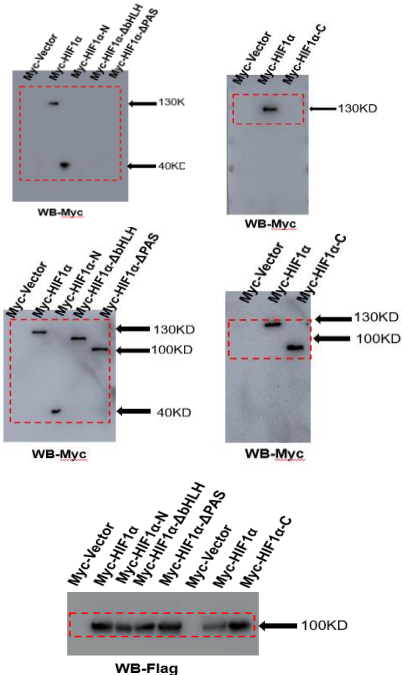

**Figure6**

**A**

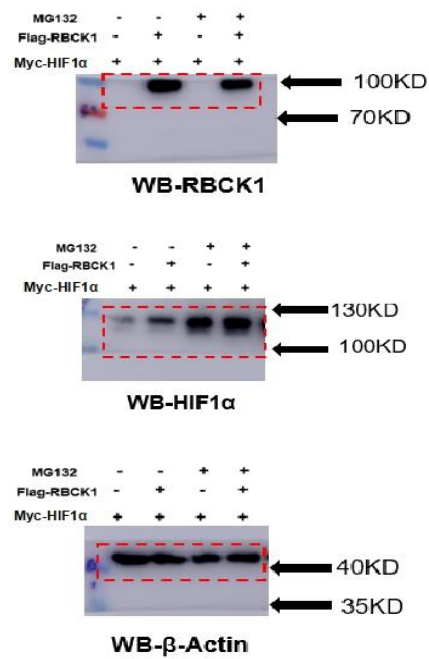

**B**

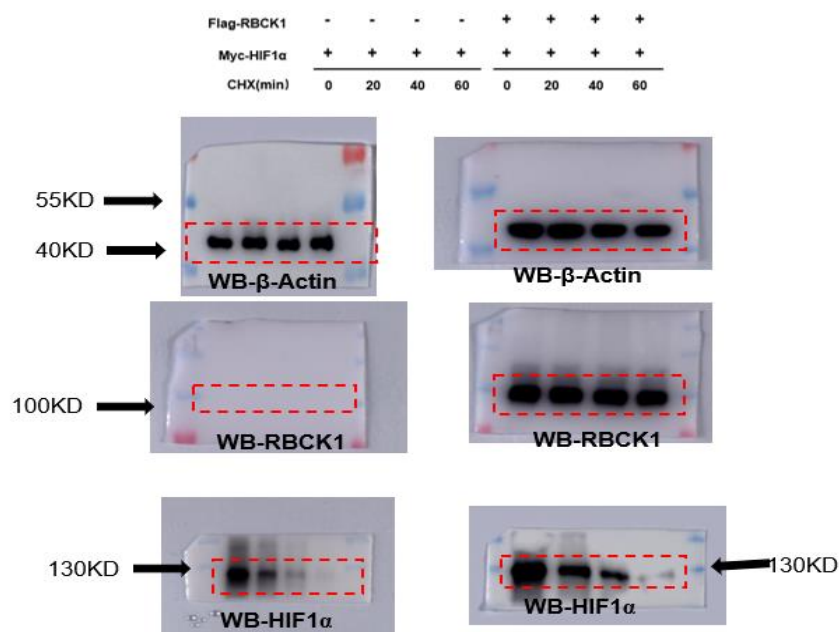

**D**

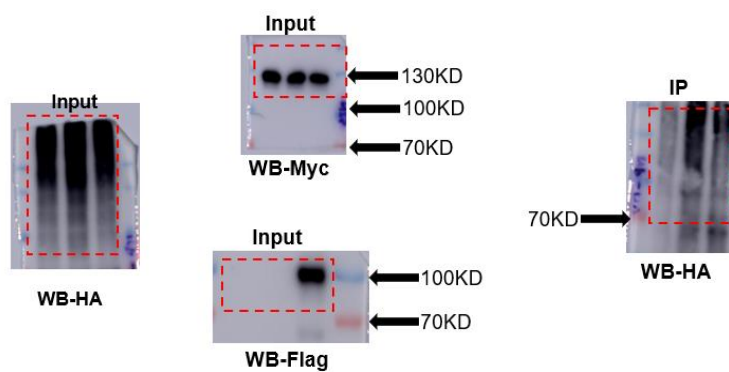

**E**

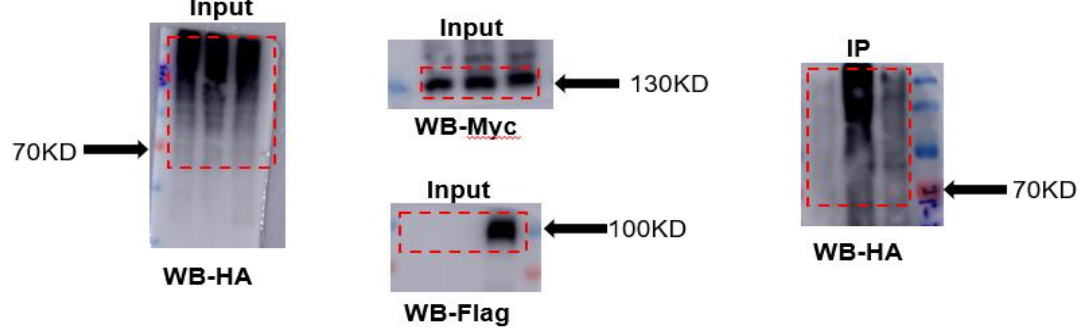

**F**

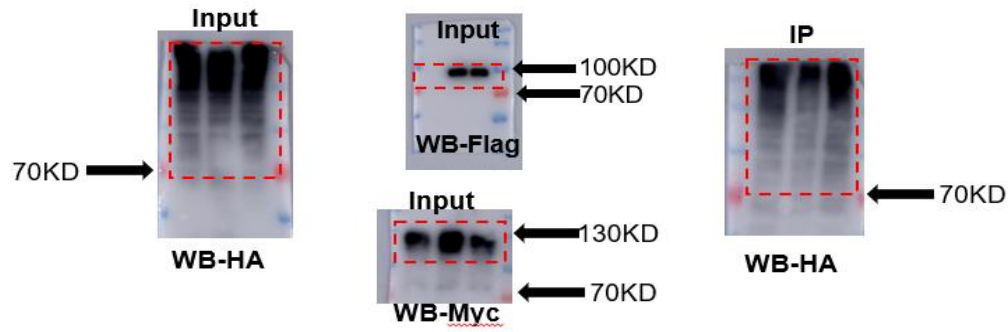

**G**

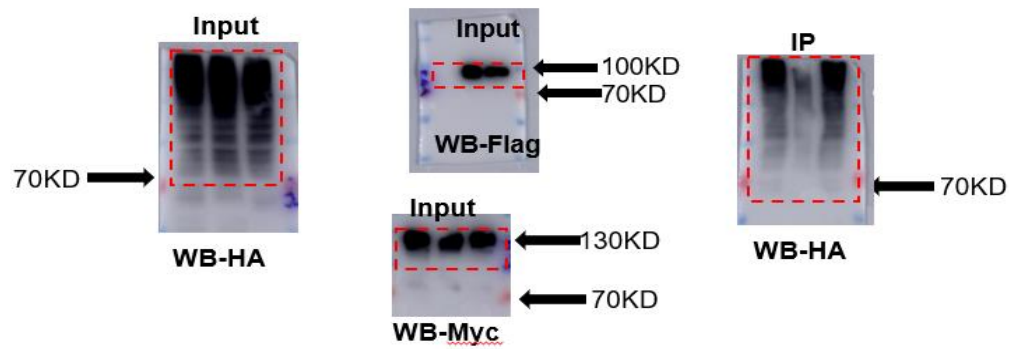

**H-K**

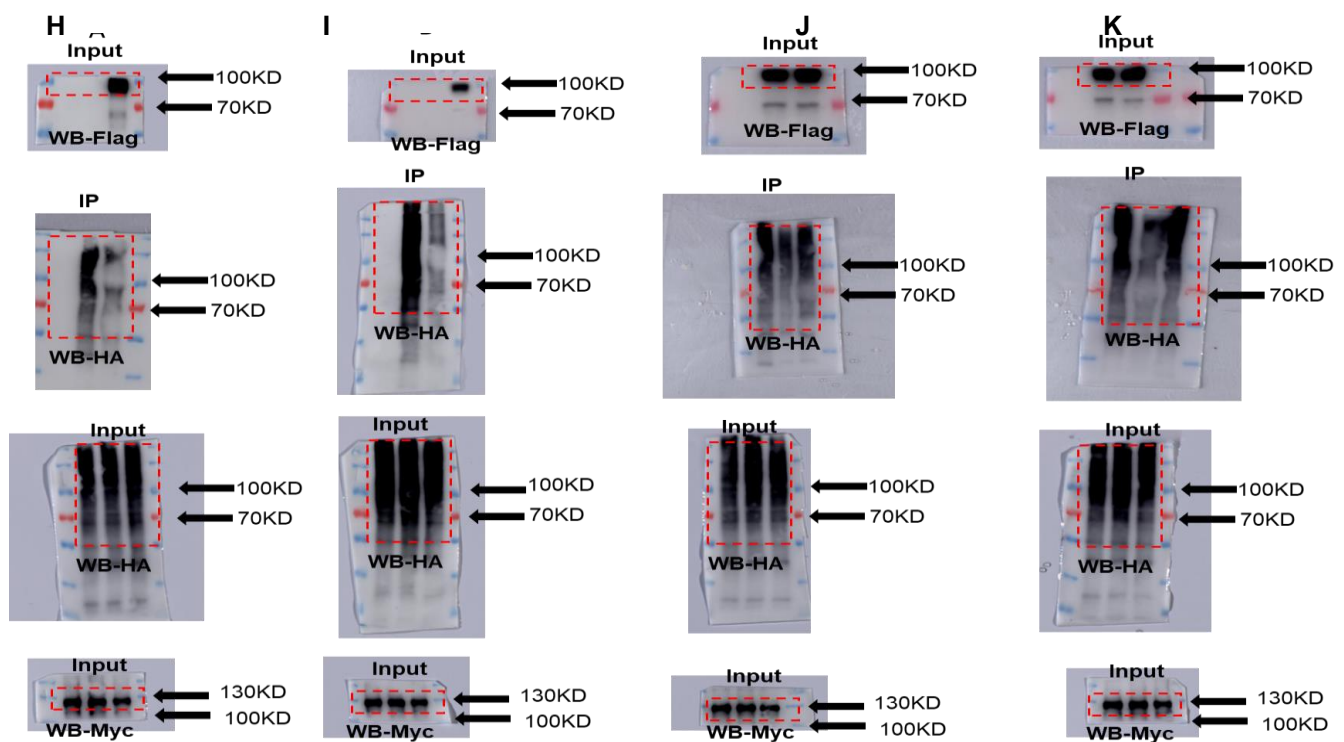

Supplement: Supplementary file 2 — Supplemental Material-WB [file 41419_2022_5473_MOESM2_ESM.pdf]
