# Supplementary material for: RBCK1 regulates the progression of ER-positive breast cancer through the HIF1α signaling
Source: Cell Death Dis. 2022 Dec 6;13(12):1023. doi: 10.1038/s41419-022-05473-6 (PMC9726878; doi:10.1038/s41419-022-05473-6)
Supplement: Supplementary file 3 — Original Data File [file 41419_2022_5473_MOESM3_ESM.pdf]

Figure1

A-B

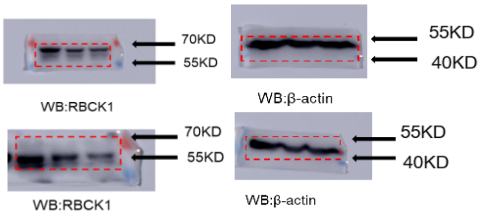

C

Relative mRNA expression

siControl			siRBCK1#1			siRBCK1#2		
1	0.98	0.96	0.36	0.29	0.32	0.43	0.47	0.53

D

Relative mRNA expression

siControl			siRBCK1#1			siRBCK1#2		
0.98	0.99	0.96	0.39	0.45	0.51	0.4	0.51	0.44

E

Relative L-Lactate Level

siControl			siRBCK1#1			siRBCK1#2		
sample1	sample2	sample3	sample1	sample2	sample3	sample1	sample2	sample3
1.01	0.99	1.00	0.39	0.41	0.43	0.59	0.59	0.60

F

Relative L-Lactate Level

siControl			siRBCK1#1			siRBCK1#2		
sample1	sample2	sample3	sample1	sample2	sample3	sample1	sample2	sample3
1.00	0.98	1.00	0.71	0.69	0.70	0.62	0.60	0.58

G

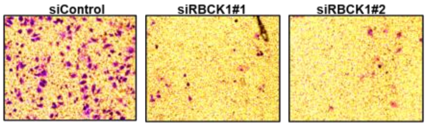

H

Migrant Cells per field

	siControl	siRBCK1#1	siRBCK1#2
sample1	224	121	68
sample2	200	104	80
sample3	210	110	90

I

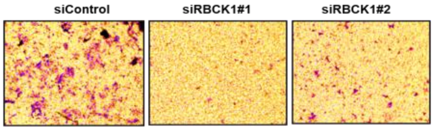

J

Migrant Cells per field

	siControl	siRBCK1#1	siRBCK1#2
sample1	204	20	73
sample2	189	36	84
sample3	210	46	80

K-L

Wound Healing Rate(%)

Time (h)	siControl			siRBCK1#1			siRBCK1#2		
	sample1	sample2	sample3	sample1	sample2	sample3	sample1	sample2	sample3
0	0	0	0	0	0	0	0	0	0
48	100	93	97	50	53	46	40	33	38

M-N

Wound Healing Rate(%)

Time (h)	siControl			siRBCK1#1			siRBCK1#2		
	sample1	sample2	sample3	sample1	sample2	sample3	sample1	sample2	sample3
0	0	0	0	0	0	0	0	0	0
48	62	65	67	35	31	36	38	35	40

O-P

Relative cell number

siControl			siRBCK1#1			siRBCK1#2		
sample1	sample2	sample3	sample1	sample2	sample3	sample1	sample2	sample3
0.95	1.05	1	0.23	0.19	0.25	0.37	0.48	0.46

Q-R

Relative cell number

siControl			siRBCK1#1			siRBCK1#2		
sample1	sample2	sample3	sample1	sample2	sample3	sample1	sample2	sample3
0.91	1.09	1.01	0.42	0.36	0.38	0.40	0.36	0.32

T

Tumor volume （mm3）

Time(days)	shControl					shRBCK1				
	sample1	sample2	sample3	sample4	sample5	sample1	sample2	sample3	sample4	sample5
0	0	0	0	0	0	0	0	0	0	0
14	82	91	83	97	96	15	24	33	21	13
21	186	243	171	215	201	29	51	49	52	20
28	390	460	360	473	422	61	83	123	86	43
35	793.15	914.24	722.56	980.82	824.95	108.65	210.00	178.61	170.00	93.81

U

Tumor weight （g）

	shControl					shRBCK1				
	sample1	sample2	sample3	sample4	sample5	sample1	sample2	sample3	sample4	sample5
Weight(g)	0.71	0.76	0.67	0.95	0.91	0.2	0.18	0.14	0.11	0.1

Figure2A
T47D

Normoxia | Hypoxia

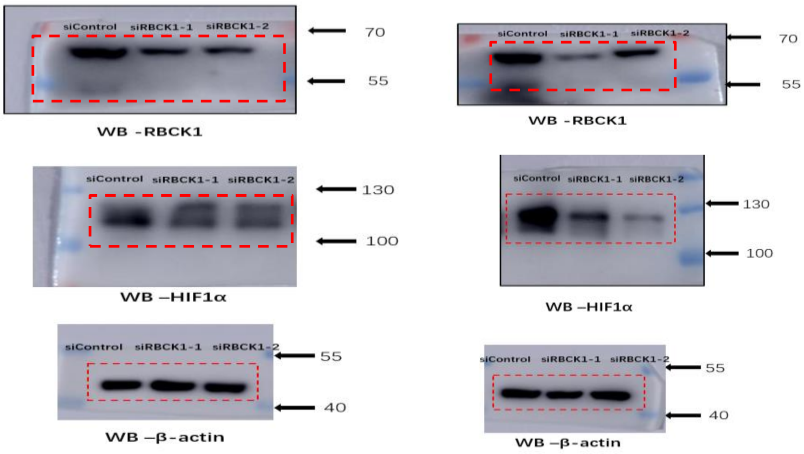

Figure2B
MCF-7

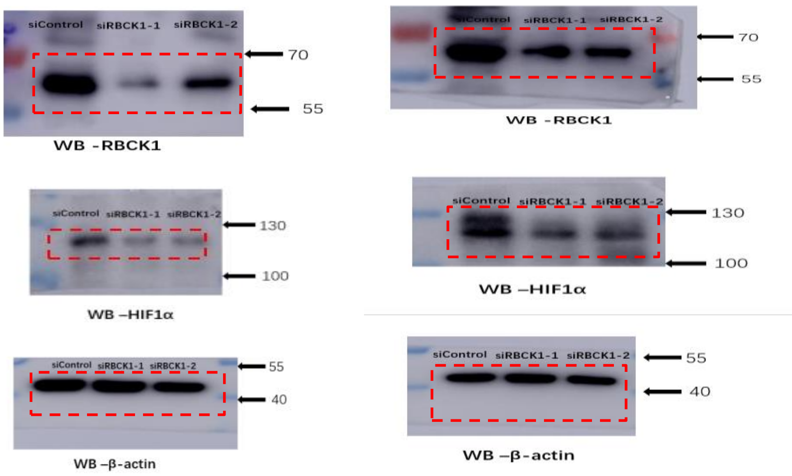

C

T47D	HIF1α Relative luciferase activity							
siControl			siRBCK1#1			siRBCK1#2		
0.95	1.00	0.98	0.09	0.07	0.10	0.41	0.34	0.38

D

MCF-7	HIF1α Relative luciferase activity							
siControl			siRBCK1#1			siRBCK1#2		
1.10	1.03	0.97	0.59	0.63	0.58	0.47	0.38	0.50

E

Relative mRNA expression

T47D		sample1	sample2	sample3
VEGFA				
Normoxia	siControl	1.072	0.928	0.985
	sRBCK1#1	0.994	1.334	1.074
	siRBCK1#2	0.911	0.836	0.956
Hypoxia	siControl	3.433	3.833	3.768
	siRBCK1#1	1.681	1.531	1.724
	siRBCK1#2	0.797	1.164	1.166
SL2AC1				
Normoxia	si-Control	1.093	0.907	0.922
	si-RBCK1#1	1.076	1.780	1.973
	si-RBCK1#2	0.879	0.825	1.007
Hypoxia	si-Control	9.574	10.198	10.123
	si-RBCK1#1	3.061	3.377	4.135
	si-RBCK1#2	3.771	4.786	4.301
PKM2				
Normoxia	si-Control	1.007	1.164	1.120
	si-RBCK1#1	0.865	0.968	1.071
	si-RBCK1#2	0.779	0.817	0.817
Hypoxia	si-Control	2.561	2.420	2.141
	si-RBCK1#1	0.889	1.135	1.328
	si-RBCK1#2	1.144	1.459	0.941

F

		Relative mRNA expression		
MCF-7		sample1	sample2	sample3
VEGFA				
Normoxia	si-Control	1.000	1.383	0.766
	si-RBCK1#1	0.594	0.314	0.272
	si-RBCK1#2	0.377	0.440	0.503
Hypoxia	si-Control	9.924	11.431	11.838
	si-RBCK1#1	3.748	4.117	3.933
	si-RBCK1#2	1.332	1.302	1.271
SL2AC1				
Normoxia	si-Control	1.000	1.000	1.022
	si-RBCK1#1	0.860	0.604	0.831
	si-RBCK1#2	0.637	0.682	0.745
Hypoxia	si-Control	9.641	9.169	8.996
	si-RBCK1#1	4.089	5.000	4.710
	si-RBCK1#2	2.905	4.282	3.659
PKM2				
Normoxia	si-Control	1.000	1.000	1.101
	si-RBCK1#1	0.851	0.753	0.949
	si-RBCK1#2	1.341	1.110	1.266
Hypoxia	si-Control	2.724	2.377	2.030
	si-RBCK1#1	0.541	0.684	0.716
	si-RBCK1#2	0.642	0.924	1.006

Figure3

A

siControl+Myc-tag | siRBCK1#1+Myc-tag | siRBCK1#1+Myc-HIF1α | siRBCK1#1+Myc-HIF1α MUT

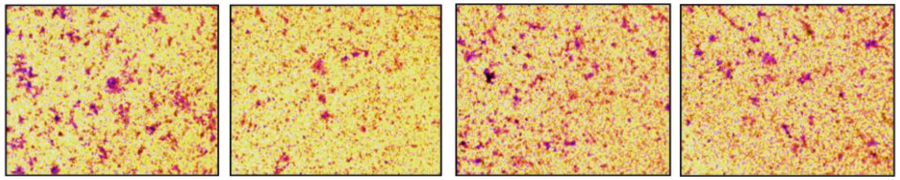

B

Migrant Cells per field

si-Control			si-RBCK1#1			si-RBCK1#1+HIF1α			si-RBCK1#1+HIF1αMUT		
201	189	187	46	32	36	140	123	106	120	138	126

C

Relative L-Lactate Level

siControl			siRBCK1#1			siRBCK1#1+HIF1α			siRBCK1#1+HIF1αMUT		
1.00	0.98	0.99	0.70	0.71	0.70	1.18	1.22	1.26	1.14	1.20	1.26

D-E

Wound Healing Rate(%)

Time (h)	si-Control			si-RBCK1#1			si-RBCK1#1+HIF1α			si-RBCK1#1+HIF1αMUT		
0	0	0	0	0	0	0	0	0	0	0	0	0
48	96	95	100	60	55	53	77	88	86	73	82	90

F-G

Relative cell number

siControl			siRBCK1#1			siRBCK1#1+HIF1α			siRBCK1#1+HIF1αMUT		
0.99	1.05	1.14	0.36	0.34	0.32	0.97	0.84	0.82	0.92	0.79	0.76

Figure4

C

Relative mRNA expression

	siControl			siRBCK1#1			siRBCK1#2		
	**sample1**	**sample2**	**sample3**	**sample1**	**sample2**	**sample3**	**sample1**	**sample2**	**sample3**
RBCK1	0.998381	0.975589	0.986029	0.451784	0.385547	0.411784	0.42442	0.365755	0.385362
VEGFA	0.97935	0.968713	0.951937	0.406839	0.338478	0.377507	0.139149	0.118716	0.105132
SLC2A1	0.993104	0.962069	0.944827	0.195565	0.153853	0.187363	0.063957	0.061558	0.075295
PKM2	0.96757	0.981073	0.981356	0.388121	0.385354	0.334326	0.284161	0.250471	0.299764
LOX10	0.987432	0.993568	0.963637	0.057719	0.05467	0.033949	0.030647	0.018534	0.019878
BNIP3	0.995281	0.981466	0.972076	0.319124	0.292158	0.290254	0.106154	0.084933	0.088653
CXCR4	0.973402	0.980658	0.972035	0.425829	0.464093	0.450374	0.21656	0.165397	0.146241

Figure5

A

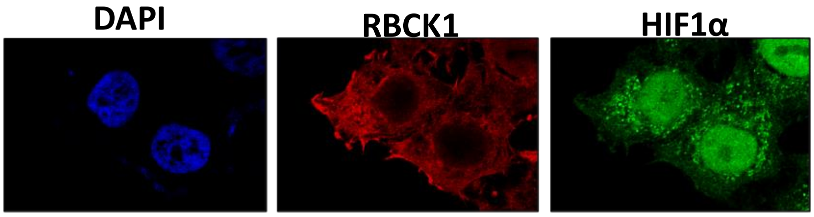

B

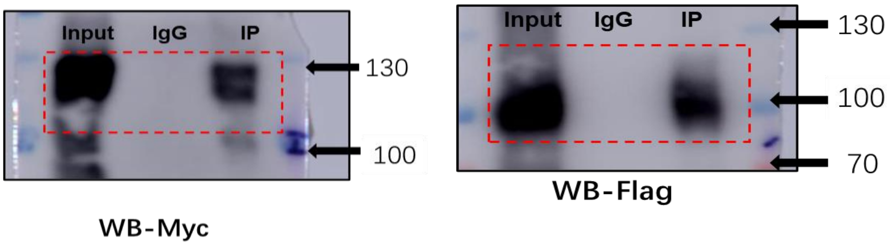

D

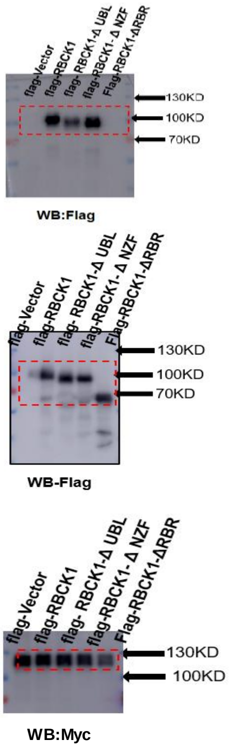

E

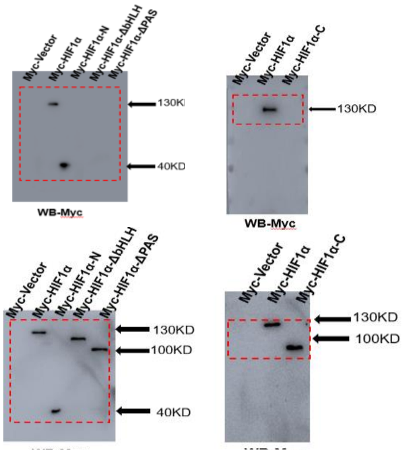

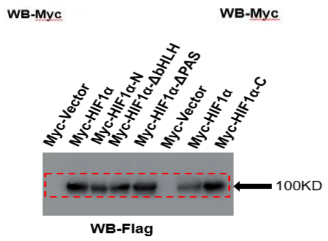
WB-Myc
WB-Myc
Myc-Vector
Myc-HIF1α
Myc-HIF1α-N
Myc-HIF1α-ΔbHLH
Myc-HIF1α-ΔPAS
Myc-Vector
Myc-HIF1α
Myc-HIF1α-C
100KD
WB-Flag

Figure6

A

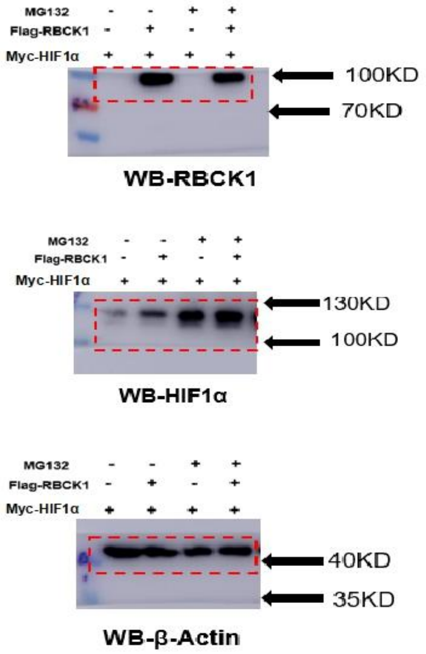

B

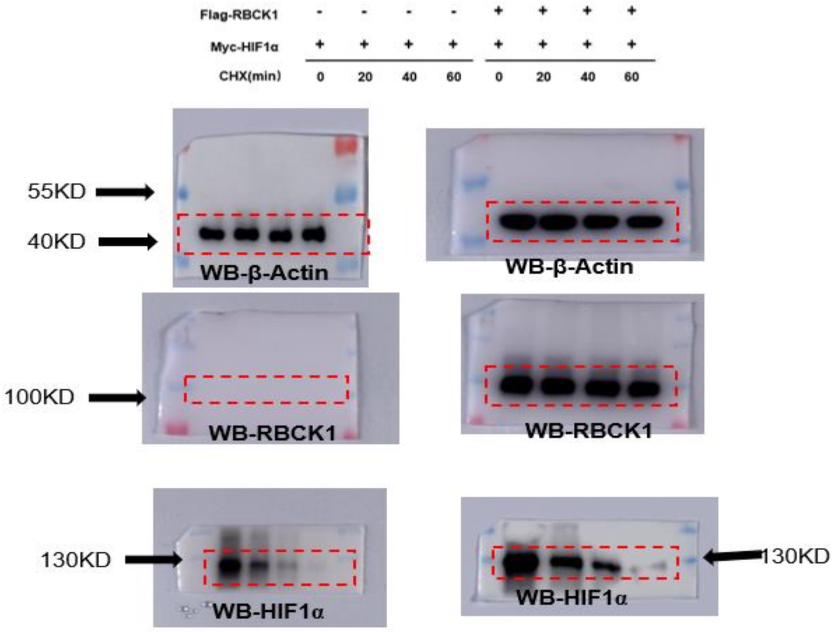

C

Relative Gray density

CHX(min）	Flag-tag+Myc-HIF1α			Flag-RBCK1+Myc-HIF1α		
0	1	1	1	1	1	1
20	0.3	0.38	0.33	0.66	0.7	0.74
40	0.2	0.1	0.15	0.5	0.43	0.53
60	0.1	0.05	0.11	0.2	0.25	0.33

D

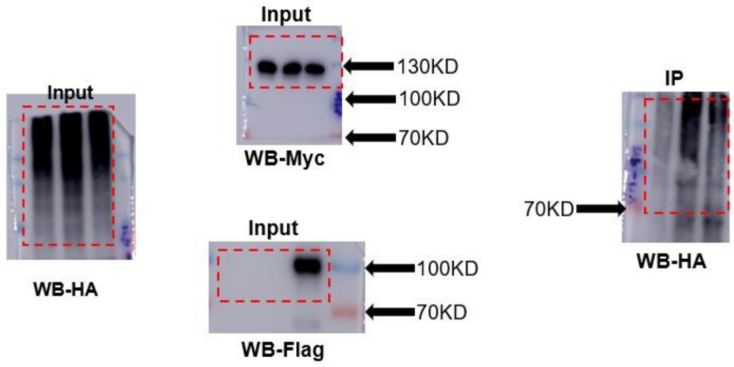

E

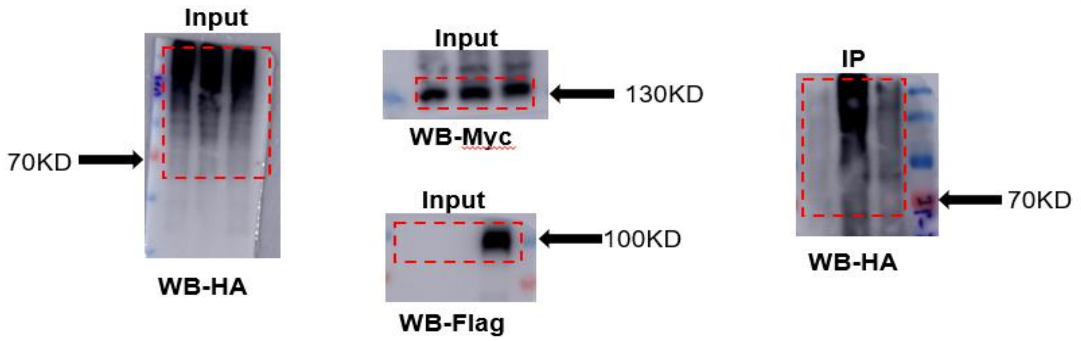
Input
70KD
WB-HA
Input
130KD
WB-Myc
Input
100KD
WB-Flag
IP
70KD
WB-HA

F

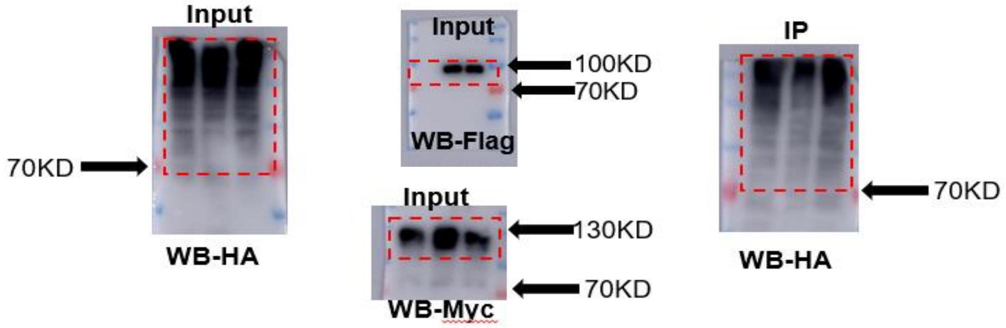
Input
70KD
WB-HA
Input
100KD
70KD
WB-Flag
Input
130KD
70KD
WB-Myc
IP
70KD
WB-HA

G

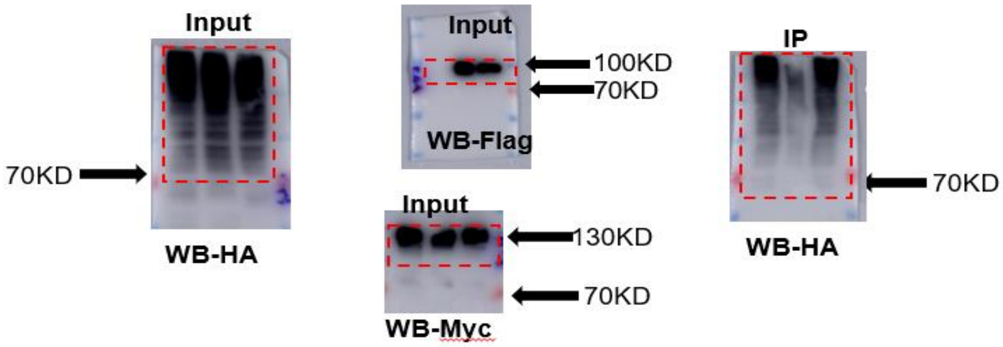
Input
70KD
WB-HA
Input
100KD
70KD
WB-Flag
Input
130KD
70KD
WB-Myc
IP
70KD
WB-HA

H-K

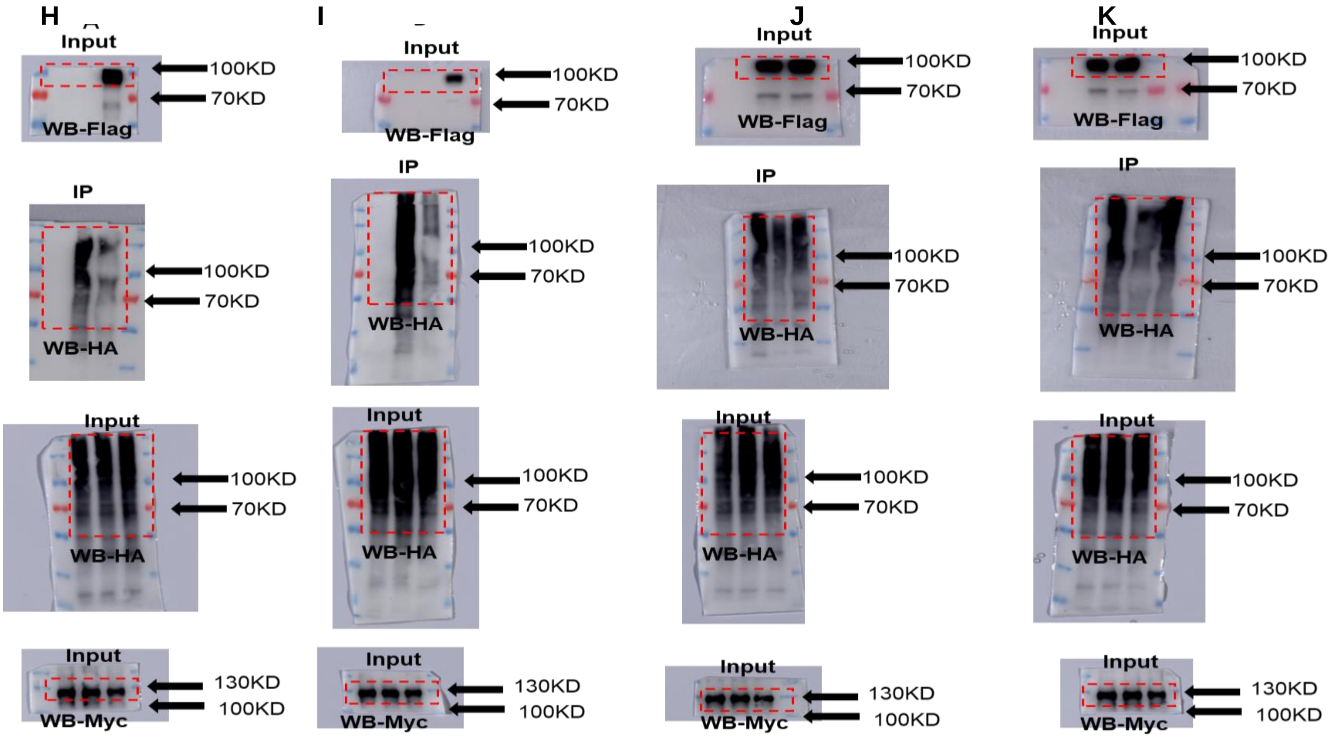
H
I
J
K
Input
WB-Flag
100KD
70KD
IP
WB-HA
100KD
70KD
Input
WB-HA
100KD
70KD
Input
WB-Myc
130KD
100KD
